# Coacting enhancers can have complementary functions within gene regulatory networks and promote canalization

**DOI:** 10.1371/journal.pgen.1008525

**Published:** 2019-12-12

**Authors:** Leslie Dunipace, Zsuzsa Ákos, Angelike Stathopoulos

**Affiliations:** California Institute of Technology, Pasadena, CA, United States of America; New York University, UNITED STATES

## Abstract

Developmental genes are often regulated by multiple enhancers exhibiting similar spatiotemporal outputs, which are generally considered redundantly acting though few have been studied functionally. Using CRISPR-Cas9, we created deletions of two enhancers, *brk5’* and *brk3’*, that drive similar but not identical expression of the gene *brinker* (*brk*) in early *Drosophila* embryos. Utilizing both *in situ* hybridization and quantitative mRNA analysis, we investigated the changes in the gene network state caused by the removal of one or both of the early acting enhancers. *brk5’* deletion generally phenocopied the gene mutant, including expansion of the BMP ligand *decapentaplegic* (*dpp*) as well as inducing variability in amnioserosa tissue cell number suggesting a loss of canalization. In contrast, *brk3’* deletion presented unique phenotypes including dorsal expansion of several ventrally expressed genes and a decrease in amnioserosa cell number. Similarly, deletions were made for two enhancers associated with the gene *short-gastrulation* (*sog*), *sog*.*int* and *sog*.*dist*, demonstrating that they also exhibit distinct patterning phenotypes and affect canalization. In summary, this study shows that similar gene expression driven by coacting enhancers can support distinct, and sometimes complementary, functions within gene regulatory networks and, moreover, that phenotypes associated with individual enhancer deletion mutants can provide insight into new gene functions.

## Author summary

Genes expressed during development are often regulated by multiple cis-regulatory sequences, non-coding DNA, also known as enhancer sequences. Many instances have been found where two or more distinct enhancer sequences support similar spatiotemporal outputs relating to a single gene. These enhancers have generally been considered redundantly acting, or overlapping in activity, though few have been studied functionally. We created deletions of coacting enhancer pairs associated with the genes *brinker* (*brk*) and *short-gastrulation* (*sog*) from the genome of *D*. *melanogaster* fruit fly using CRISPR-Cas9 genome editing. Surprisingly, opposite phenotypes relating to some target genes are associated with the enhancer deletions. Deletion of one enhancer generally exhibits phenotypes in early embryo patterning similar to respective gene mutants; whereas, in contrast, deletion of the other presents unique phenotypes including change in cell number for a particular tissue in the embryo, the amnioserosa. In summary, this study shows that coacting enhancers driving similar expression outputs can support distinct, and sometimes complementary, functions to differentially impact the development of embryos and that the individual mutation of these enhancers can provide insight into new gene functions.

## Introduction

It has been demonstrated that many developmental genes are associated with a number of enhancers that support similar or overlapping spatiotemporal gene expression patterns, termed “shadow” or “sibling” enhancers [1,2]. To provide insight into their roles, studies of these coacting cis-regulatory elements have ranged from assay of individual enhancer activity in the context of small reporter genes to whole-genome approaches in which conservation of sequence was used as a proxy for function. The first study that coined the term shadow enhancer focused on two genes in *Drosophila* embryos, *brinker* (*brk*) and *short gastrulation* (*sog*), which are each associated with two enhancers that control their expression in the early embryo [1]. These enhancers drive expression in overlapping domains when evaluated by reporter assay, suggesting that their roles are either redundant or overlapping in function. Subsequently, studies of such coacting enhancer pairs associated with other genes have found that individual enhancers within a pair are conditionally required, as single enhancer mutants are viable under normal conditions but both enhancers are required to support robustness under adverse conditions, such as genetic perturbation or stressful environmental conditions [e.g. 3,4,5]. On the other hand, a whole genome study of shadow enhancers found that these coacting sets of enhancers exhibit higher sequence conservation suggesting they also likely have some non-redundant functions [6].

In *Drosophila*, large reporter constructs allow assay of the role of individual enhancers in supporting gene expression in the context of the intact cis-regulatory systems. For example, large reporter constructs were used in live imaging experiments to determine that at some loci, such as *knirps*, coactive enhancers can function in an additive way in terms of gene output; while at other loci (*hunchback* in anterior regions, and *snail*), the function of enhancers is sub-additive: as the combined activity of the two-acting enhancers assayed is less than the sum of each individual enhancer [7,8]. Similarly, the proper expression domains of a gene are also dependent on the cooperation between coacting enhancer pairs, requiring both enhancers for precise patterning [2,9]. Therefore, coacting enhancers pairs can either synergize or compete to regulate levels or spatial output of gene expression.

For the gene *brinker* (*brk*), it appears that the two enhancers regulating expression in the early embryo are co-active for only a short period of time [10]. The 5’ enhancer (*brk5’*, also known as 5’CRM or *brkNEE*), located ~10 kB upstream of the promoter, acts first to support expression in a thin lateral stripe along the dorsal-ventral (DV) axis and the 3’ enhancer (*brk3’* also known as 3’CRM or *brk* shadow), located ~10 kB downstream of the promoter, acts subsequently to support expression in a broad lateral stripe [1,11–13]. Through deletion analysis in the context of large reporter constructs, our previous analysis showed that the *brk5’* and *brk3’* enhancers act in series and that the switch is regulated by a promoter proximal element [10]. Furthermore, the *brk* gene expression pattern changes in time from a thin lateral stripe to one that is broad [14,15], presumably, relating to sequential action of the *brk5’* and *brk3’* enhancers. As a result of this previous study, we hypothesized that the spatiotemporal change in *brk* gene expression supported by serial action of these two enhancers, though subtle, might relate to different roles of Brk protein, which functions as a transcriptional repressor [e.g. 14]. Nevertheless, it was left untested in previous studies how *brk* early embryonic enhancers individually impact Brk-dependent functions, as the large reporter constructs did not preserve Brk function but instead the gene was replaced by reporter gene *gfp*.

New advances in genome editing present the opportunity to functionally examine roles for individual enhancers in native context. Here we conducted an analysis of phenotypes associated with enhancer mutants for *brk* as well as *sog*, created using CRISPR-Cas9 [rev. in 16] to delete these sequences from the endogenous locus, and assayed their individual and combined roles to provide insight into the function of coacting enhancers.

## Results

### Deletion of *brk* early embryonic enhancers supports viability but uncovers amnioserosa defect

**Fig 1:**
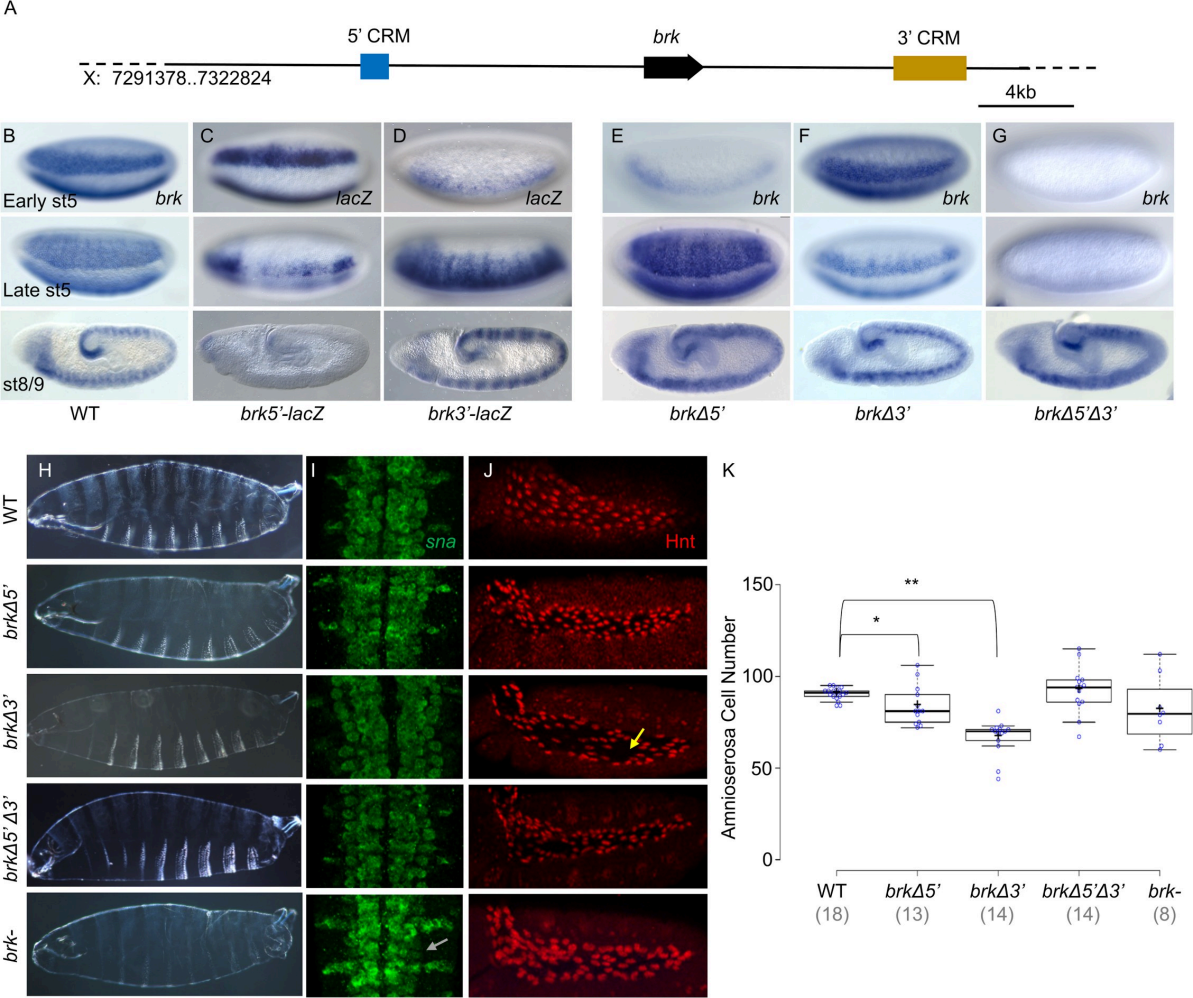
Pre-gastrulation expression of *brk* is not required for viability but deletion of the *brk3’* enhancer leads to a loss of amnioserosa specification. (A) Genomic locus of *brk* indicating the location of the *brk5’* and *brk3’* enhancers relative to the *brk* gene coding sequence. (B-E) In situ hybridization using *brk* (B,E-G) or *lacZ* (C,D) riboprobes detected by alkaline phosphatase (AP) on (B) wildype (WT), (C) *brk5’-lacZ* reporter, (D) *brk3’-lacZ* reporter, (E) *brk Δ5’*, (F) *brk Δ3’*, and (G) *brk Δ5’Δ3’* embryos. In this and all subsequent figures, unless otherwise indicated, all embryos are lateral view with anterior to the left and dorsal on top. The top row shows the narrow lateral expression of *brk* during early stage 5 that is primarily driven by the *brk5’* enhancer. The middle row shows the broad lateral pattern of *brk* expression at late stage 5 which is lost when the *brk3’* enhancer is deleted. The bottom row are stage 8 or 9 embryos which all express *brk* in the ectoderm. (H) Dark field images of lateral view of cuticle preps from first instar larvae. Only the *brk-* gene mutant embryos show a loss of ventral denticle bristles. (I) Fluorescent *in situ* hybridization (FISH) against *sna* riboprobe in the ventral neurogenic ectoderm of stage 9–11 embryos, anterior up and posterior down. The WT and *brk* CRISPR-Cas9 mutants all show three rows of *sna* expressing central nervous system (CNS) cells on either side of the midline with 2–3 peripheral nervous system (PNS) cells per segment extending out laterally. Only the *brk-* gene mutant embryos show a loss of the third row of *sna* expressing cells in the CNS (grey arrow). (J) Lateral view of stage 10–13 embryos immunostained for Hnt to mark the amnioserosa cells. Gaps in the *brk Δ3’* amnioserosa marked with yellow arrow. (K) Number of amnioserosa cells on the lateral side of the embryo for each of the genotypes. The number of amnioserosa is significantly different from WT in the *brkΔ5’* embryos (* P = 0.03, Welsh’s t-test) and the *brkΔ3’* (** P = 1.5x10^-7^, Welsh’s t-test), but not in the *brkΔ5’Δ3’ or brk-* (P = 0.8 and P = 0.2, respectively, Welsh’s t-test) For this and subsequent box plots, the center lines show the medians; box limits indicate the 25th and 75th percentiles as determined by R software; whiskers extend 1.5 times the interquartile range from the 25th and 75th percentiles, outliers are represented by dots; crosses represent sample means; data points are plotted as open circles. Number of embryos counted shown under genotype.

To start, we used CRISPR-Cas9 to delete two enhancers, *brk5’* and *brk3’*, from the native *brk* locus (Fig 1A). Surprisingly, flies containing deletion of either enhancer individually (*brkΔ5’* or *brkΔ3’*) or both together (*brkΔ5’Δ3’*) are viable and therefore embryos obtained from homozygous mutant stocks were characterized. Expression supported by these enhancers, through *lacZ* gene reporter assay (Fig 1C and 1D;[10]), was compared with resulting *brk* gene expression in mutant embryos containing deletion of one or both enhancers. Deletion of the enhancer located upstream (*brkΔ5’*) leads to a loss of the early narrow pattern (early st5 Fig 1E, compare with 1B); deletion of the enhancer located downstream (*brkΔ3’*) leads to a loss of the later, broad pattern (late st5 Fig 1F); and deletion of both *brk*5’ and *brk*3’ enhancers (*brkΔ5’Δ3’*) leads to a complete loss of *brk* expression in the cellularized embryo at stage 5 (Fig 1G). These observations agree with a previous analysis where deletions of these enhancers were made within a large reporter construct in which the *brk* coding sequence was replaced with *gfp* [10]. Additionally, the deletions made at the native locus permit extension of the earlier study by allowing analysis of phenotypes resulting from perturbed *brk* expression. When viability at the embryonic stage specifically was examined (see Methods), we found that there was a slight increase in embryonic lethality compared to wildtype in each of the single mutant embryos but, surprisingly, more than 85% of embryos were viable (S1A Fig).

To provide insight into how these enhancer sequences support Brk function, we examined embryonic phenotypes linked to *brk-* gene mutants. Previous studies of *brk-* gene mutant phenotypes had suggested that early *brk* expression is responsible for defective patterning of the ventral ectoderm at stage 9 [17]. This change presents as a reduction in *snail* gene (*sna*)*-*expressing neuroblasts as well as a loss of ventral denticle bristles in the cuticle in the ventral neurogenic region (*brk-*, Figs 1H, 1I and S1B). However, the early embryonic *brk* enhancer deletions had little to no effect on either neuroblast specification (Fig 1I) or denticle band formation (Figs 1H and S1B). We observed that in all three enhancer mutant lines *brk* expression is restored by stage 8/9 (st8/9; Fig 1E–1G). Therefore, other enhancer(s) likely support Brk’s role at later stages. Furthermore, as *brk-* gene mutants are zygotically lethal but embryonic enhancer deletions are not, *brk* gene activity at a later stage of development likely is required to support viability [18]. Consequently, these early embryonic enhancer deletions allowed us to analyze the specific contribution of Brk activity in the cellularizing embryo to later phenotypes.

Previous studies have shown that specification of amnioserosa, an extraembryonic tissue that serves as a structural support during germ band elongation and retraction during embryo morphogenesis [19], is not affected in *brk* gene mutants [17]. Using Hindsight (Hnt) antibody to label amnioserosa cells, we confirmed that neither the *brk-* gene mutant nor the *brkΔ5’Δ3’* enhancer deletion mutant has significantly different amnioserosa cell numbers compared to wildtype (Fig 1J and 1K). We did, however, observe higher variability in cell number in these two genotypes compared to wildtype (Fig 1K; Levene’s test for equality of variance (Levene’s): *brkΔ5’Δ3’* P = 0.02; *brk-* P = 0.007). In contrast, amnioserosa cell number in *brkΔ3’* embryos is significantly lower than wildtype with large empty areas visible within the field of amnioserosa cells (Fig 1J arrow, Fig 1K; Levene’s: brkΔ3’ P = 0.04). Amnioserosa cell number in the *brkΔ5’* is also significantly different from wildtype, being lower on average, but additionally more variable with some embryos having more cells than wildtype (Fig 1J and 1K, brkΔ5’; Levene’s: P = 0.005). These findings indicate that *brk* does affect amnioserosa specification. As BMP signaling, specifically high level output via ligand Decapentaplegic (Dpp), is thought to directly relate to amnioserosa specification [20], these results also suggest *brk* expression impacts high level Dpp signaling outputs. The fact that amnioserosa is a transient structure, undergoing cell death after germ band retraction [19], helps to explain how the variability in amnioserosa cell number observed in these enhancer mutants can be tolerated and compatible with embryo viability. Nevertheless, these different amnioserosa phenotypes associated with individual enhancer deletions reveal new and distinct roles for Brk in supporting early embryonic patterning that could not be identified using *brk-* gene mutants (see Discussion).

The unexpected fact that the *brkΔ5’Δ3’* embryos had milder defects than the single enhancer deletions hinted at a compensatory mechanism and led us to investigate the effects of *brk* driven by the individual enhancers within the gene network of the early embryo.

### Both enhancers are required to properly regulate canonical Brk target genes’ early expression and to establish *dpp* dosage

**Fig 2:**
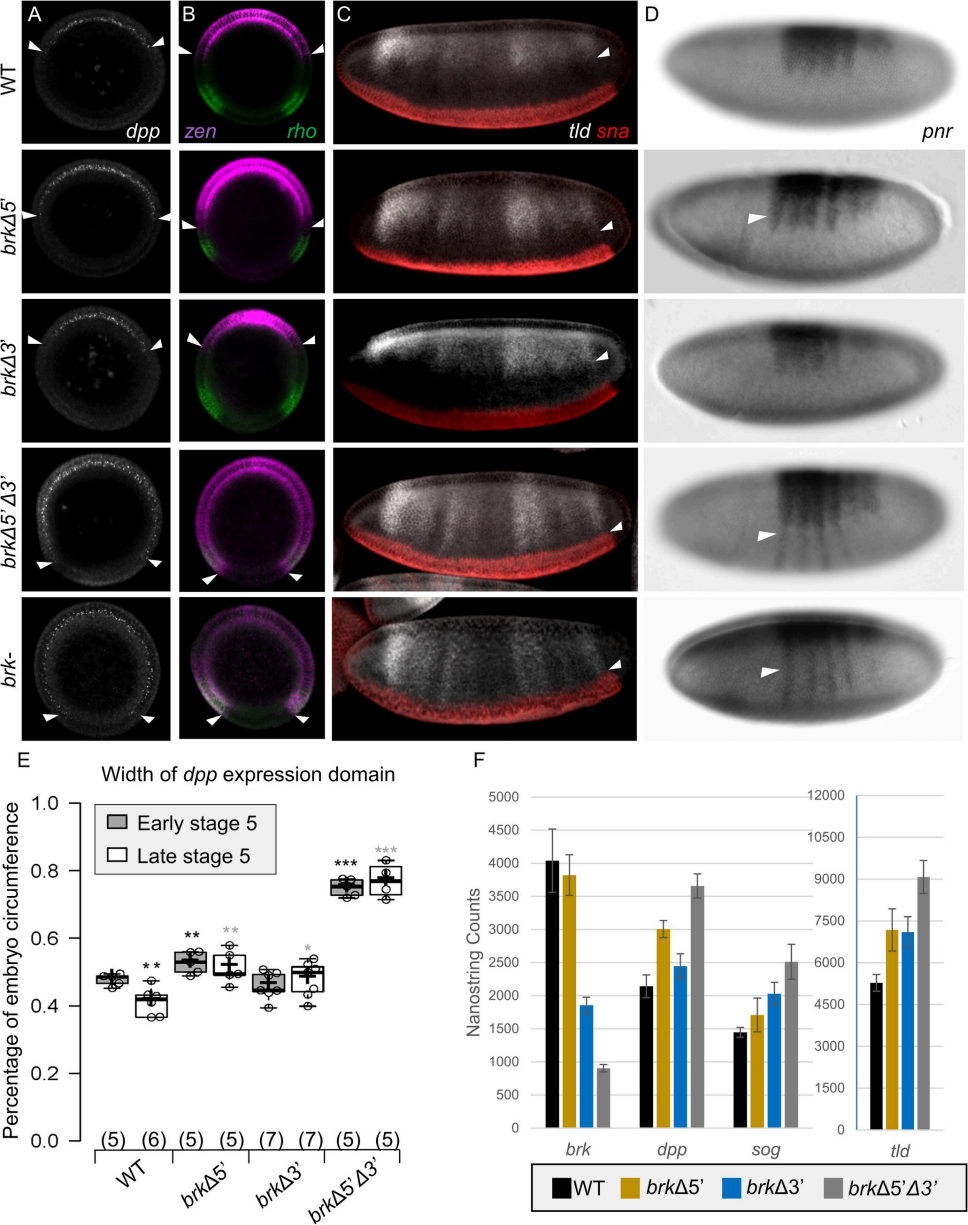
Proper expression of *brk* at early stage 5 is required for setting the ventral boundary of *dpp* and BMP target genes. (A-B) FISH using an (A) intronic *dpp* probe (white) or (B) *zen* (purple) and *rho* (green) probes on manually chopped cross-sections of late stage 5 embryos. White arrowheads indicate the ventral most cell expressing *dpp* (A) or *zen* (B) on either side of the embryo. (C) Early stage 6 embryos with riboprobes to *sna* (red) and *tld* (white). The ventral extent of *tld* expression is indicated with white arrowheads. (D) AP detection of *pnr* riboprobe on early stage 6 embryos. Expansion of the lateral stripes towards the ventral side of the embryo is indicated with white arrowheads. (E) Quantification of the percentage of embryo circumference expressing *dpp* in early stage 5 and late stage 5 embryos. The percentage of the embryo circumference expressing *dpp* was quantified using 5–7 cross-sectioned embryos per stage of each genotype (see Materials and methods). Mutant genotypes were compared to WT embryos of the same stage. Black asterisk indicates that the measurement is significantly different from the early stage 5 WT embryo (P = 0.005 late stage 5 WT, P = 0.008 *brkΔ5’*, P = 0.2 *brkΔ3’*, P<0.00001 *brkΔ5’Δ3*’), and grey asterix indicates the same for the late stage 5 embryos (P = 0.002 *brkΔ5’*, P = 0.02 *brkΔ3’*, P<0.0001 *brkΔ5’Δ3*’; indicated in the graph as *P<0.05, **P<0.01, ***P<0.0001, Student’s t-test). (F) NanoString results for *brk*, *dpp*, *sog*, and *tld* in nc14C (late stage 5) embryos (see Materials and methods). For this and all subsequent NanoString graphs, the results are given in arbitrary units and the SEM for 6 (WT, *brk Δ5’Δ3’*) or 5 (*brk Δ3’*, *brk Δ5’*) individual embryos are shown by black bars.

It has been shown that direct repression by Brk is important to establish the proper expression pattern of the *dpp* gene as well as several Dpp target genes [14,20]. The most studied of these Brk/Dpp target genes are *zen*, *tld*, and *pnr*. We therefore looked at the expression pattern of these four genes in each of the *brk* gene and enhancer mutant backgrounds (see Fig 2A–2D). *brkΔ5’Δ3’* mutant embryo phenotypes were consistent with *brk-* gene mutants with the *dpp*, *zen*, *pnr* and *tld* expression domains expanded all the way to the dorsal border of *snail* (*sna*), on the ventral side of the embryo. *brkΔ5’* mutants show a milder defect, but similar trend, with all four target genes slightly expanded. The *brkΔ3’* mutants, upon first observation show no clear expansion of any of these early target genes.

However, upon closer scrutiny, all of the mutants, including *brkΔ3’*, have an effect on *dpp* when expression dynamics are taken into consideration (see Fig 2E). The wildtype expression domain of *dpp* encompasses about 47% of the embryo circumference at early stage 5, and by late stage 5 is refined to around 41%. In the *brkΔ3’* embryos, the extent of the *dpp* expression domain is not significantly different from wildtype at early stage 5; however, it fails to refine at late stage 5. Collectively, these data demonstrate that *brk* is required both for the proper establishment of the *dpp* expression domain in early stage 5 (*brk5’* primary) as well as the refinement of the domain throughout stage 5 (*brk3’* primary) to regulate *dpp* dosage and impact BMP signaling at stage 6.

However, amnioserosa specification relies on input from Dpp but also on other genes. The lack of refinement in *brkΔ3’* mutants is not likely to explain why amnioserosa cell number is low in this mutant background, specifically. We turned toward analysis of other genes acting to support patterning in order to determine if any changes were specific to loss of the *brk3’* enhancer that could explain why amnioserosa cell counts are low in this particular mutant background.

### NanoString data supports the view that some genes are regulated by *brk* activity driven by both enhancers whereas others are regulated predominantly by the *brk3’* enhancer activity

We conducted a quantitative analysis of target gene RNA levels using NanoString technology in order to provide further insight into the role of individual *brk* enhancers in the regulation of gene expression [21]. The NanoString system permits precise quantification of transcripts across five orders of magnitude from a single embryo without the need to fragment, amplify, or reverse transcribe the RNA. In a previous study, we used expression of ~70 genes representing pivotal patterning genes active in early embryos to assay the dorsal-ventral patterning gene regulatory network state in the early embryo [22]. This method facilitates assay of temporally-precise samples, which for *Drosophila* embryos is challenging as embryonic development cannot be synchronized within larger collections.

**Fig 3:**
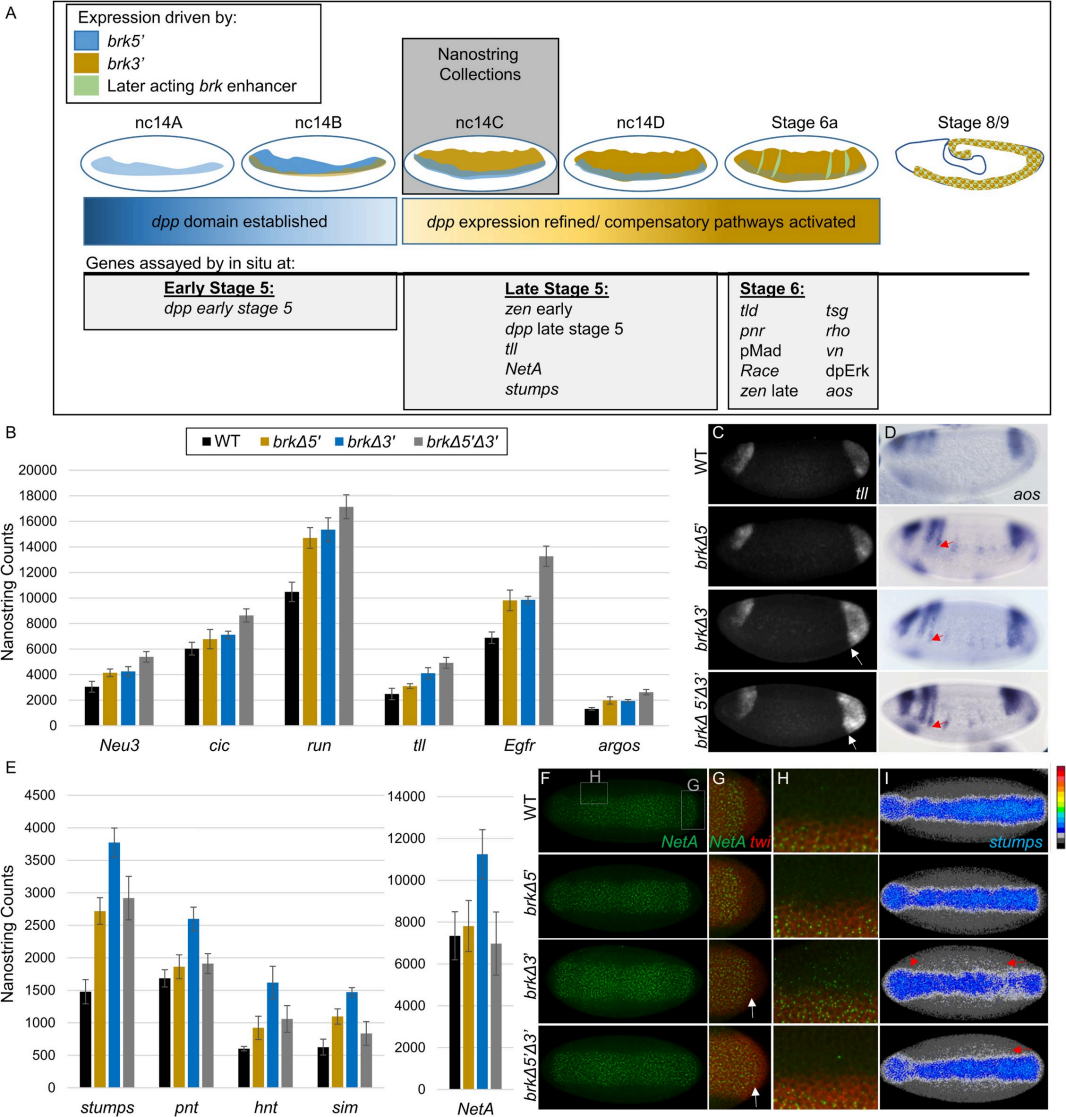
NanoString uncovers both additive and *brk3’* enhancer-specific gene expression effects. (A) Illustration of changing contribution of the activity driven by the *brk3’* (blue), *brk5’* (gold), and later acting *brk* enhancers (green) to the *brk* expression pattern during early stages of development. All embryos for NanoString were collected at nc14c. Genes assayed by *in situ* hybridization in this study are listed under the developmental stage in which they were analyzed. (B) Graphical representation of NanoString results from nc14C embryos showing genes in which there is an additive effect with the highest level of expression in the *brk Δ5’Δ3’* embryos. (C) FISH staining using *tll* riboprobe on late stage 5 embryos. Expansion of *tll* expression in posterior indicated by white arrows. (D) AP detection of *aos* riboprobe on early stage 6 embryos. Red arrows mark ventral-most expression of *aos* head stripe in each genotype. (E) NanoString targets that show a significant upregulation in the *brkΔ3’* embryos. (F-I) Ventral view of late stage 5 embryos hybridized to riboprobes for (F) intronic *NetA*, (G,H) intronic *NetA* and *twi*, or (I) *stumps*. (G) is a magnified view of the posterior of the embryo shown in (E) showing *NetA* (green) expression as well as *twi* (red) expression to mark the mesoderm; expansion into the posterior marked by white arrows. Similarly (H) is a magnified image of the mesoderm boundary towards the anterior of the embryo. The expression of *stumps* (I) has been false colored with a heatmap (scale shown to the right with red highest and black lowest expression, see Materials and methods). Areas of expanded expression indicated with red arrows.

Individual embryos of wildtype or *brk* enhancer mutant backgrounds were assayed for gene expression profiles at one timepoint during late stage 5 [nuclear cycle (nc) 14C] when both enhancers should be active (Fig 3A, see Materials and methods). Quantitative mRNA data was compared for four genotypes: wildtype (WT: *yw)*, *brkΔ5’*, *brkΔ3’*, and *brkΔ5’Δ3’*. First, as proof of principle, total counts of *brk* mRNA were measured and, as expected, the *brkΔ5’* data is similar to wildtype (as the *brk3’* enhancer is predominantly acting at late stage 5), whereas counts are decreased for the *brkΔ3’* and *brkΔ5’Δ3’* datasets (Fig 2F). The *brkΔ5’Δ3’* counts are the lowest, supporting the view that the *brk5’* enhancer is partially active at this stage as well. Despite the lack of detectable staining in these mutants at nc14C, however, the NanoString counts for *brkΔ5’Δ3’* are non-zero. This could indicate that there is low level maternal, ubiquitous transcript in these embryos or that a different enhancer drives low-level expression that we are unable to visualize by *in situ*.

The NanoString approach serves as a sensitive, quantitative method to investigate how gene expression supported by individual enhancers may have additive (i.e. phenotypes of increasing severity) or antagonistic effects (i.e. opposite phenotypes) on target gene expression outputs. NanoString allows us to detect mRNA abundance, which is complementary to the spatial data provided by stainings. For example, *dpp* levels show a consistent trend between area of expression, visualized by *in situ* hybridization (Fig 2A and 2E), and levels of expression as measured by NanoString (Fig 2F). During late stage 5, the *brkΔ5’Δ3’* mutant has both the highest level and the broadest domain of *dpp* expression. The *brkΔ5’* mutant also shows increased *dpp* levels compared to wildtype, though not as high as the double deletion, consistent with the partial spatial expansion of the *dpp* expression domain seen in this mutant (Fig 2F, compare with 2A and 2E). The *brkΔ3’* embryos show a *dpp* expression level similar to, if slightly increased over wildtype, in agreement with the lack of refinement of the *dpp* expression domain between early and late stage 5 in this genotype (Fig 2F, compare with 2A and 2E). However, for some other Brk targets, we observe a lack of correlation between levels and expression domain. For example, *tld* expression is equivalently increased in both the *brkΔ5’* and *brkΔ3’* embryos according to the NanoString data, although we observe spatial expansion of expression only in *brkΔ5’* embryos (Fig 2F, *tld*, compare with 2C). The parallel analysis of gene expression on both area (*in situ* hybridization) and level (NanoString) of expression allowed us to more fully investigate the changes in gene regulation in these mutant embryos (Fig 3A).

*brk* encodes a transcription factor that acts as a transcriptional repressor to limit BMP signaling and support patterning in the early embryo and at other stages of development including in the wing disc [11,14]. In this role, it has been shown that *brk* functions redundantly with *short gastrulation* (*sog*), a homolog of chordin in vertebrates, which encodes another inhibitor of signaling acting in the extracellular space [17,23]. As both Brk and Sog regulate BMP signaling outputs activated by Dpp ligand, we hypothesized that *sog* may be able to compensate for compromised *brk* enhancer function in early embryos. Indeed, late stage 5 expression of *sog* is affected in *brk* enhancer mutants (Fig 2F). The *brkΔ5’* embryos show near wildtype levels, but the *sog* expression is significantly increased in the *brkΔ3’* and *brkΔ5’Δ3’* embryos. These compensatory changes in *sog* levels may act to rescue decreases in *brk* and serve as a partial explanation for the viability of embryonic *brk* enhancer mutants (see Discussion).

Among the set of patterning genes studied using NanoString, a small set appeared to respond in what appears to be an additive manner, in terms of target gene expression phenotype severity, in *brk* enhancer mutants. Individual deletions each result in slight expression changes which are amplified in *brkΔ5’Δ3’* embryos (Fig 3B). This set of genes is diverse in both the expression domains as well as the pathways in which they function. They include *Neu3* (*Meltrin*), which encodes a metalloprotease and is expressed in a pattern largely overlapping with *brk*, as well as more broadly-expressed genes such as *capicua* (*cic*) and *runt* (*run*) which encode transcription factors [24–26]. Even anterior-posterior regulated genes, such as the transcription factor *tailless* (*tll*) [27], are upregulated, and this change can be seen as *tll* expression is visibly expanded posteriorly in *brkΔ3’* embryos and even more so in the *brkΔ5’Δ3’* embryos (Fig 3C, white arrows). NanoString also highlighted two known *brk* targets from the EGFR pathway, *Egfr* and *argos* (*aos*) *[28]*. *aos* shows a similar additive regulatory trend, with slight expansion of the anterior stripes in the *brkΔ5’*, slightly more derepression in this region in the *brkΔ3’*, and full expansion of these stripes towards the ventral side of the embryo in *brkΔ5’Δ3’* (Fig 3D, red arrows).

While our analysis identified no genes that show significant upregulation in *brkΔ5’* compared to the other genotypes (S2 Fig), a number were significantly upregulated in the *brkΔ3’* embryos including several genes that are ventrally-expressed in the embryo, within the presumptive mesoderm (Fig 3E). The gene *NetA* [29] shows the largest difference in expression levels in the *brkΔ3’* embryos, as compared to the other backgrounds (Fig 3E). Lateral expression of *NetA* outside of the presumptive mesoderm (marked by *twi* expression) is expanded in *brkΔ3’* embryos, especially in the anterior third of the embryo (Fig 3F and 3H). In addition, the expression domain of *NetA* is expanded in the posterior in both the *brkΔ3’* and the *brkΔ5’Δ3’* embryos (Fig 3G, arrows). This posterior expansion tracks with *brkΔ3’* and suggests that late Brk expression supported by the 3’ enhancer may be required for terminal patterning (i.e. posterior *NetA* expansion relates to an indirect effect). Another ventrally-expressed gene, *stumps*, also appears expanded in lateral regions specifically in embryos mutant for *brk3’* (Fig 3I, red arrows). These data indicate a previously undescribed role for *brk* in which proper expression of *brk* in late stage 5, which is primarily supported by *brk3’*, is required for setting the dorsal boundary of some ventrally expressed genes.

To determine if these observed phenotypes relate to direct action by Brk, we examined published Brk chromatin immunoprecipitation data [ChIP-seq; 28] for two different timepoints, 2–2.5 hr and 3–3.5 hr, roughly corresponding to early and late stage 5, respectively, and representing primarily *brk5’-* (2–2.5 hr) or primarily *brk3’-* (3–3.5 hr) driven *brk* expression. These data show Brk binding at the *NetA* and *stumps* loci primarily during late stage 5 but not significantly during early stage 5 [28], supporting the view that the *brk3’*-driven *brk* expression is responsible for regulating these genes (S3 Fig). Notably, though, in both *NetA* and *stumps*, we see a greater overall increase in expression (Fig 3E) and visible expansion into the neuroectodermal tissue in the trunk of the embryo in the *brkΔ3’* compared to the *brkΔ5’Δ3’* (Fig 3F, 3H and 3I). This lack of expansion in the double deletion could be an indirect effect of the loss of *brk* expression driven by the two early acting enhancers, possibly relating to the expanded domain of *dpp* expression in these embryos that could either directly or indirectly repress the expression of these ventrally expressed genes. Again, we find an instance where the double enhancer deletion exhibits a more mild defect than the single deletion.

In summary, these NanoString data support the view that Brk transcription factor activity driven by the *brk3’* enhancer is exclusively required to regulate a subset of genes, and demonstrate that the *brk5’* and *brk3’* enhancers have important, nonredundant functions during development. Furthermore, as a set of genes exhibits comparable gene expression in *brkΔ5’* and *brkΔ5’Δ3’* mutants but distinct outcomes in the *brkΔ3’* mutant (Fig 3E), the *brk3’* enhancer may function to balance action of the *brk5’* enhancer. Loss of the *brk3’* enhancer makes a difference only if the *brk5’* has acted first to establish a wildtype gene regulatory environment and suggests these two enhancers act in a complementary manner.

### Differential BMP target gene expression in *brk* mutants results from differences in gene regulatory network state in these mutants

Nanostring as well as *in-situ* analysis of the *brk* mutants revealed varying expression of effectors and known regulators of BMP signaling (Figs 2A–2F and S2). Similarly, we observed differential specification of amnioserosa cells, which are specified by peak BMP signaling, in the three mutant lines (Fig 1J and 1K). Therefore, we analyzed BMP signaling and resulting target gene expression in these mutants to assess the impact on the broader gene regulatory network.

**Fig 4:**
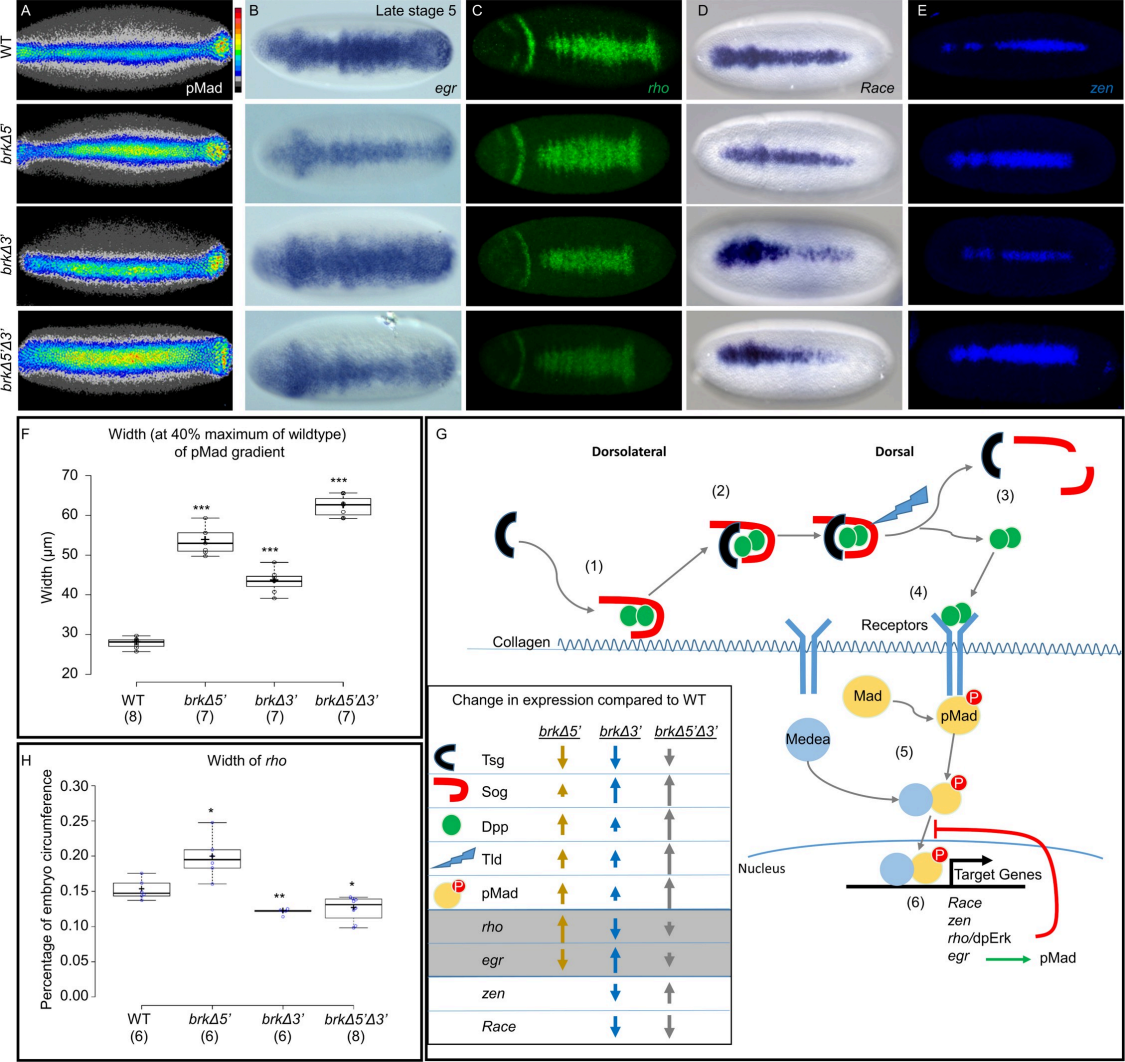
pMad gradient is expanded in *brk* mutants but BMP target gene expression at late stage 6 does not correspond. (A) Dorsal view of early stage 6 embryos immunostained with pMad antibody. Z-projection is false colored with a heatmap (key indicated to the right, red highest, black lowest expression, see Materials and methods). (B-E) Dorsal view of FISH (B,D) or AP stained (C,E) *in situs* against riboprobes to (B) *egr*, (C) *rho*, (D) *Race*, and (E) *zen*. All embryos are stage 6 unless otherwise noted. (F) Quantification of pMad gradient. Width was measured at 40% maximum of WT pMad gradient (see Materials and methods). Width of the pMad gradient is significantly different from WT in the *brk Δ5’* (Welch’s *t* test: t = 18.8, df = 7.4, ***P<0.0001), in the *brkΔ3’* (Welch’s *t* test: t = 13.0, df = 8.0, ***P<0.0001) and in the *brk Δ3’*,*brkΔ5’* embryos (Welch’s *t* test: t = 31.3, df = 8.4, ***P<0.0001). (G) Graphic representation of some of the key effectors and target genes expressed through BMP signaling in the early embryo. (1) On the dorso-lateral side of the embryo Dpp dimers form a complex with Sog, bound to collagen; Tsg binds to this complex and releases it from the collagen matrix so that it can (2) diffuse dorsally. (3) Tld cleaves Sog and releases Dpp from the complex so that it can (4) bind to the BMP receptors and activate the phosphorylation of Mad. (5) Phosphorylated Mad binds to the co-Smad, Medea, and enters the nucleus. (6) pMad and Medea initiate transcription of target genes, some of which have either positive (*egr*) or negative (*rho*/EGFR signaling) feedback into BMP signaling. The changes in gene expression of key effectors and target genes in each of the mutants compared to WT as determined by *in situ* and/or NanoString data is depicted by arrows. (H) Box plot of width of *rho* expression domain measured as percentage of the embryo circumference. (see Materials and methods) (P = 0.01 *brkΔ5’*, P = 0.002 *brkΔ3’*, P = 0.0048 *brkΔ5’Δ3*’; in graph indicated by *P<0.01, **P<0.005 Welsh’s t test).

Bmp signaling is regulated at many levels: from the production and transport of the ligand to the dorsal midline, binding and activation of the receptors, and finally the phosphorylation of Mad and its import into the nucleus to initiate transcription of target genes [rev. in 30,31]. Once produced, the transport to and concentration of Dpp at the dorsal midline requires a set of proteins including Sog, Twisted gastrulation (Tsg), and Tld [32]. Sog forms a complex with the ligands, Dpp and Scw, aided by Collagen IV which can act as a scaffold for BMP-Sog binding (Fig 4G (1), [33]). The binding of Tsg to this complex releases it from the Collagen IV and allows for its movement and promotes the long range shuttling of the ligand (Fig 4G (1) and (2), [34]). Free Dpp and Scw ligands are released from the Sog-Tsg complex through cleavage of Sog by the protease Tld (Fig 4G (3), [35]). After being released from the Sog-Tsg complex, Dpp is then free to bind to and activate the BMP receptors [Fig 4G (4)], leading to the phosphorylation of Mad. In wildtype embryos Dpp activity, commonly quantified by measurement of phosphorylated Mad (pMad [36]), is restricted to a narrow peak at the dorsal midline. After phosphorylation, pMad binds to the co-Smad, Medea, and enters the nucleus where it activates target gene expression [Fig 4G (5) and (6)].

Brk can affect the steps in this pathway both directly and indirectly. We have shown that deletion of either of the two *brk* enhancers alone, or both together, have differential impacts on the production of Dpp (Fig 2E). In addition, in all the *brk* enhancer mutants, both *sog* and *tld* expression levels are increased (Fig 2F), while *tsg* is downregulated (S2 Fig) with expression in the middle of the embryo lost in the *brkΔ5’* and *brkΔ5’Δ3’* deletions (S4A Fig). To determine the effect of these changes in gene expression domains on BMP signaling within *brk* mutants, we measured the width of the pMad gradient for each mutant at early stage 6. Similar to the results for *dpp* and early *brk* target genes (Fig 2), the pMad gradient is significantly widened in *brkΔ5’* embryos (53.6 ± 4.4 μm) and in *brkΔ5’Δ3’* embryos (62.3 ± 2.7 μm) compared to the WT (27.9 ± 1.3μm) (Fig 4A and 4F). The gradient is also wider in *brkΔ3’* embryos, but to a lesser extent (43.5 ± 2.9 μm; Fig 4A and 4F). The differential effects on regulators of BMP signaling in these mutant backgrounds provides insight into the differences observed in pMad expression, as the ratio of expression levels between the functional activators (*dpp*, *tld*, and *tsg*) to the inhibitor (*sog*) is different in the three mutant backgrounds.

Several targets of BMP signaling have previously been shown to have either positive (i.e. *egr*) or negative (i.e. EGFR signaling) feedback into BMP signaling and the formation of the pMad gradient. The BMP target gene *egr* has been shown to be a necessary component in the positive feedback mechanism, which refines and amplifies the pMad expression domain [36]. Inversely, the import of pMad into the nucleus has been shown to be negatively affected by EGFR signaling. Specifically, it was found that hyperactivated EGFR signaling can directly inhibit translocation of pMad into the nucleus [37]. Notably, both *egr* and components of the EGFR signaling pathway show opposite responses in the *brkΔ5’* and *brkΔ3’* embryos.

The expression domain of two genes involved in EGFR signaling, *rhomboid* (*rho*) encoding a protease and *vein* (*vn*) encoding a ligand, are significantly expanded at the dorsal midline of the *brkΔ5’* embryos (Figs 4C and S4B). It follows that dpERK is also expanded in this mutant (S4C Fig). As these genes are known targets of BMP signaling, this result is consistent with the fact that these embryos have higher *dpp* levels and wider pMad expression domains. The negative feedback from high levels of EGFR signaling seen in the *brkΔ5’* may explain why the wider pMad gradient in these mutants fails to lead to higher target gene expression such as *zen* and *Race* (Figs 4D, 4E, S4E and S4F). The other *brk* mutants, however, do not show the same response. Despite the wider pMad gradient in *brkΔ3’*, the expression of *rho* in these embryos is significantly narrower than wildtype (Fig 4C and 4H). Unexpectedly, the *rho* and dpERK expression in the *brkΔ5’Δ3’* embryos, although slightly reduced, is the most similar to wildtype of the *brk* mutants (Figs 4C, 4H and S4C). In the case of EGFR signaling feedback, it appears as if half action by Brk is more disruptive than a loss of Brk activity (see Discussion).

Similarly, *egr* is oppositely regulated in the two single mutants. In the *brkΔ3’* mutants the *egr* expression domain is expanded; while both the domain and the levels of expression are significantly lower in the *brkΔ5’* embryos (Fig 4B). As *egr* is known to refine and concentrate the pMad gradient, this may in part explain why peak target genes, such as *zen* and *Race* (Figs 4D, 4E, S4D, S4E and S4F), are narrower or missing in the *brkΔ3’* embryos and subsequently why the amnioserosa cell number is lower in these mutants. Again, we see that the expression of *egr* in the double mutant is somewhat intermediate between the two single mutants (Fig 4B). Surprisingly, the expression of the peak target genes in the *brkΔ5’Δ3’* mutants is inconsistent, with *zen* being expanded and *Race* expression disrupted (Figs 4D, 4E, S4D, S4E and S4F).

Brk can affect so many different components of the gene network that a simple model of the known feedback and feedforward loops can not completely explain the new state created in these *brk* enhancer mutants. Furthermore, the finding that some regulators of BMP signaling are differentially affected in the *brk5’* and *brk3’* mutants provide an explanation for why BMP target gene expression varies in these genotypes, as these target gene outputs reflect an additive effect of many Brk actions (Fig 4G; see Discussion).

### Deletion of shadow enhancers associated with the *sog* gene also exhibit opposite phenotypes, distinct from the gene mutant

**Fig 5:**
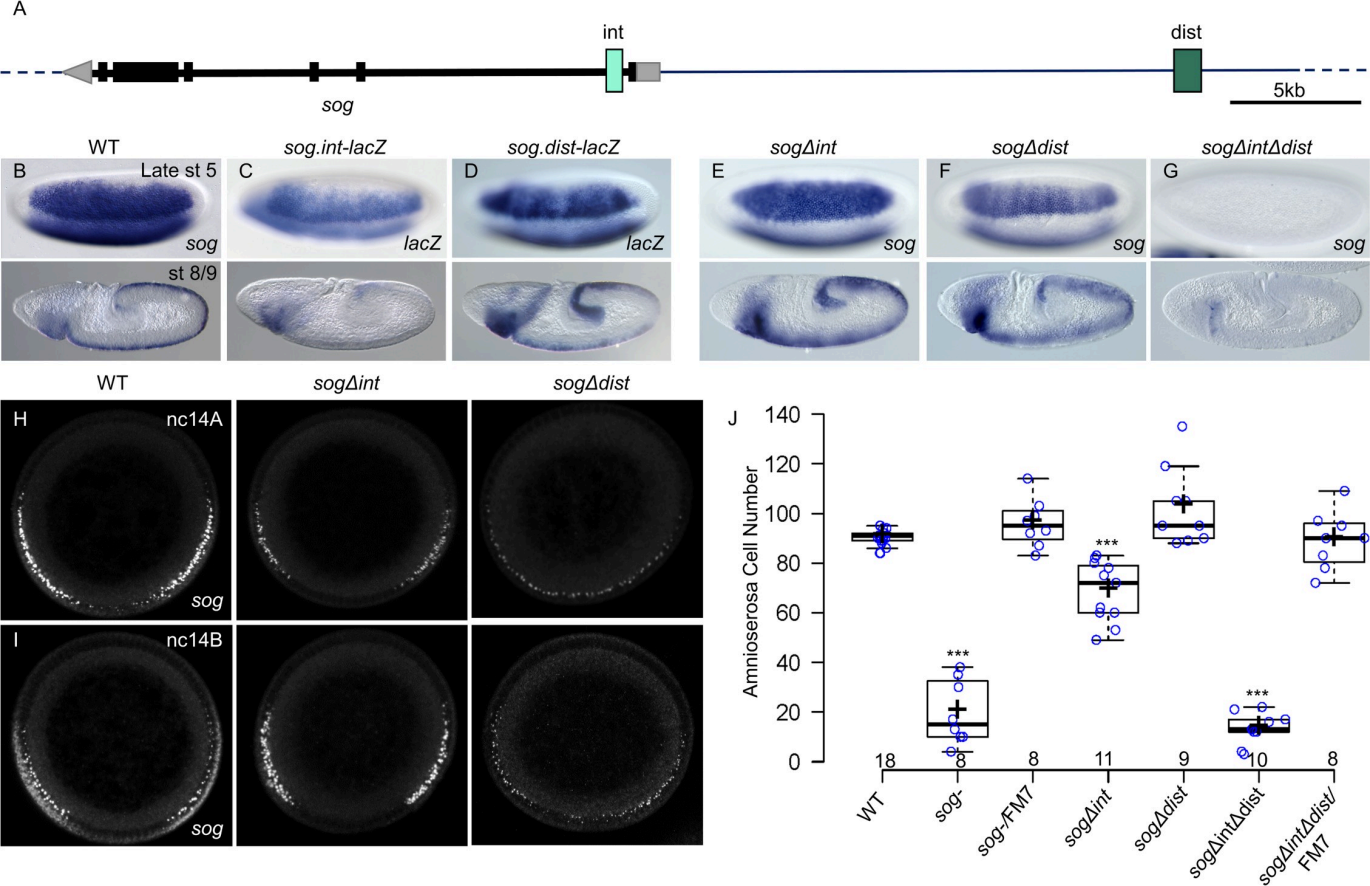
Two coacting enhancers at the *sog* locus drive largely overlapping expression domains in the early embryo but differ in expression levels and sensitivity to ventral repression, leading to opposite effects on amnioserosa specification. (A) Genomic locus of *sog* indicating the location of the *sog*.*int* (light green) and *sog*.*dist* (dark green) enhancers relative to *sog*. Exons for *sog* are shown as black boxes and 5’ and 3’ UTR indicated in grey. (B-G) *In situ* hybridization using *sog* (B,E-G) or *lacZ* (C,D) riboprobes detected by alkaline phosphatase (AP) on (B) WT, (C) *sog*.*int-lacZ* reporter, (D) *sog*.*dist-lacZ* reporter, (E) *sogΔint*, (F) *sogΔdist*, and (G) *sogΔintΔdist* embryos. The top row of embryos is late stage 5 and the bottom row is stage 8 or 9. (H,I) FISH staining on manually cross-sectioned embryos in early stage 5 at nc14A (H) or nc14B (I) with riboprobe to the first exon of sog which includes part of the 5’ UTR, thus labelling both nascent and mature transcripts. (J) Number of amnioserosa cells on the lateral side of the embryo for each of the genotypes. The number of amnioserosa is significantly different from WT in the mutant embryos for *sog-* gene (***P = 1.2x10^-6^, Welsh’s t-test), *sogΔint* (***P = 1.1x10^-4^, Welsh’s t-test), and *sogΔintΔdist* (***P = 1.3x10^-13^, Welsh’s t-test), but not for the *sog*- gene heterozygote (*sog-/*FM7), *sogΔdist*, or the *sog* enhancer double mutant heterozygotes (*sogΔintΔdist/*FM7) (P = 0.18, P = 0.05, and P = 0.78, respectively, Welsh’s t-test).

**Fig 6:**
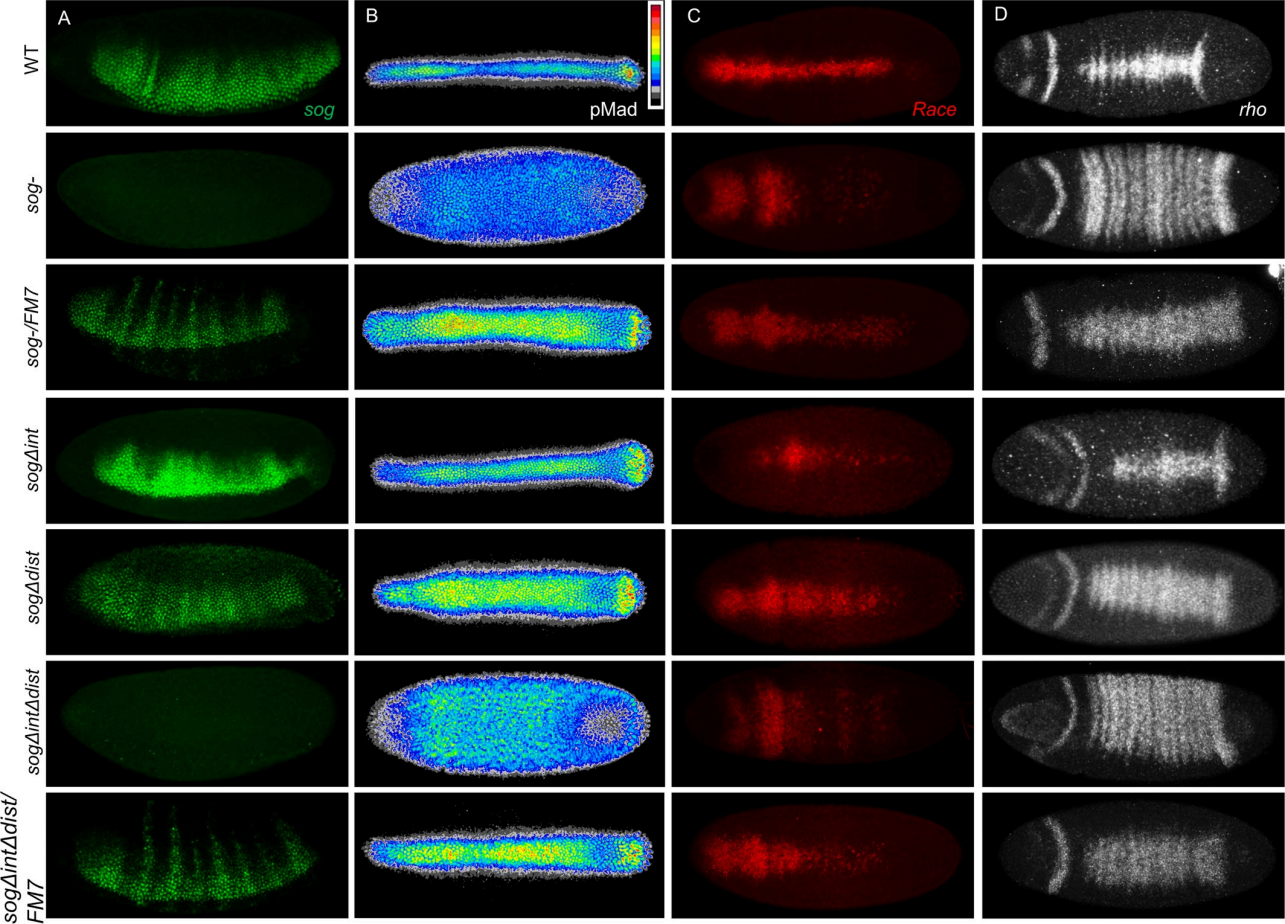
Shadow enhancers associated with the *sog* gene also exhibit opposite phenotypes relating to BMP signaling outputs. (A-D) (A) Lateral or (B-D) dorsal view of stage 6 embryos hybridized with riboprobes to (A) *sog*, (C) *Race*, (D) *rho*, or (B) immunostained with an antibody to pMad and false colored with a heatmap (key indicated to the right, red highest, black lowest expression, see Materials and methods). *sog-* is gene mutant, whereas *sogΔint*, *sogΔdist*, and *sogΔintΔdist* are deletions of individual enhancers.

We hypothesized that shadow enhancers, in general, are able to support complementary activities. To provide additional support for this idea, we deleted two early embryonic enhancers driving expression of the gene *sog*, which has also been characterized as having coacting enhancers (Fig 5A [1]). At the *sog* locus, one enhancer is located proximal to the promoter within the first intron (*sog*.*int*) whereas the other is located distally, more than 10 kB upstream (*sog*.*dist* also known as *sog*.*NEE* or *sog*.*shadow*) (Fig 5A). Both *sog*.*int* and *sog*.*dist* enhancers have been shown to support expression in early embryos within a broad lateral stripe with apparently no difference in their timing of action when examined by reporter gene assay (Fig 5C and 5D, see also [1,38,39]). Similar to the *brk* enhancer deletions, both the *sogΔint* and *sogΔdist* enhancer deletions are viable; although the double mutant, which exhibits a loss of all early embryonic expression (Fig 5G), is not viable. Unlike *brk*, however, deletion of either of the early *sog* enhancers did not lead to an obvious change in expression domain of the gene, but there was a marked difference in expression levels (Figs 5E, 5F and 6A).

Analogous to what has been described at the *snail* locus [7,8], deletion of the enhancer more proximal to the gene (*sog*.*int*) leads to an increase in expression levels while deletion of the more distant enhancer (*sog*.*dist*) leads to a decrease in expression levels. Also consistent with the findings from the *sna* locus [8], the distal enhancer is highly sensitive to repression. In wildtype embryos in nc14A, *sog* expression is expanded into the ventral most cells (Fig 5H), but is quickly excluded from the ventral region by nc14B (Fig 5I), presumably through repression by Sna [40]. In the *sogΔint* mutants, however, there is no ventral expression at all in nc14, while in the *sogΔdist* mutants this ventral expression is retained until nc14C (Fig 5H and 5I).

As we hypothesized, however, the single enhancer deletions in *sog* also show opposite phenotypes on the larger gene network. Amnioserosa cell number is decreased in the *sogΔint* while it is increased on average in the *sogΔdist* (Fig 5J). The amnioserosa cell numbers for *sogΔdist* are similar to those for a *sog-* gene heterozygous embryo (Fig 5J), further confirming the determination that the *sogΔdist* mutation decreases the levels of *sog* expression. All of the *sog* mutant backgrounds except for *sogΔintΔdist* demonstrated increased variability in amnioserosa cell number compared to wildtype (Fig 5J; Levene’s: *sog-* P = 1.0x10^-5^, *sogΔint* P = 1.0x10^-5^, *sogΔdist* P = 2.4x10^-4^, *sogΔintΔdist* P = 0.09). The increased variability suggests that, like Brk, Sog also functions to canalize BMP peak signaling output.

To further characterize these mutants, we looked at the indicator of BMP signaling (pMad) as well as a peak BMP target gene (*Race*) and a mid-level BMP target gene (*rho*). In the *sog-* gene mutant, pMad is present at uniform low levels in an expanded area encompassing the dorsal half of embryos, in contrast to the narrow band of graded expression present in wildtype embryos (Fig 6B). However, in both *sog* enhancer mutants, pMad staining is expanded compared to wildtype (Fig 6B). Previous observations and modeling works have shown that BMP signaling is directly affected by the dosage of *sog*. When *sog* is expressed at lower levels, such as in a heterozygous *sog* mutant embryo, the pMad expression domain is significantly wider than wildtype (Fig 6B); conversely, the peak of pMad signal is narrower and lower when an extra copy of Sog is introduced [41]. The levels of *sog* are visibly lower in the *sogΔdist* mutant compared to wildtype (Fig 6A), and the resulting pMad pattern is more broad, consistent with heterozygous *sog-* gene mutant embryos (Fig 6B). In contrast, levels of *sog* transcript are visibly increased at stage 6 in the *sogΔint* mutant background compared to wildtype (Fig 6A), yet the pMad expression is not narrowed compared to wildtype as one would expect from *sog* over-expression (Fig 6B). It is likely that timing, as well as levels of expression of *sog* are important for defining the pMad gradient at stage 6.

Downstream BMP target genes are also differentially affected in these mutants. In a *sog* gene mutant, expression of *Race*, a peak level target gene, is lost in the trunk but retained at the anterior, whereas expression of the mid-level target gene *rho* is expanded throughout the trunk (Fig 6C and 6D). These results are consistent with the broad low level pMad in the *sog-* gene mutant, and an absence of peak levels of BMP signaling. The *sogΔdist* enhancer mutant appears to increase BMP signaling outputs; both peak response (i.e. *Race*) as well as mid-level signaling output (i.e. *rho*) expression domains are wider, similar to the *sog-* heterozygous embryos (Fig 6C and 6D). However, deletion of *sogΔint* leads to a mostly wildtype *rho* expression pattern (Fig 6D), but a decrease in *Race* expression (Fig 6C). This is consistent with the model of *sog* overexpression where most of the Dpp ligand is sequestered and unable to activate peak response genes. Therefore, in terms of *Race* expression, the two coacting enhancers at the *sog* locus appear to support opposite functions. These results suggest that both *sog*.*int* and *sog*.*dist* enhancers are required to express the proper levels of *sog* for both peak and mid-level BMP targets to be expressed possibly through a mechanism of enhancer competition (see Discussion).

**Fig 7:**
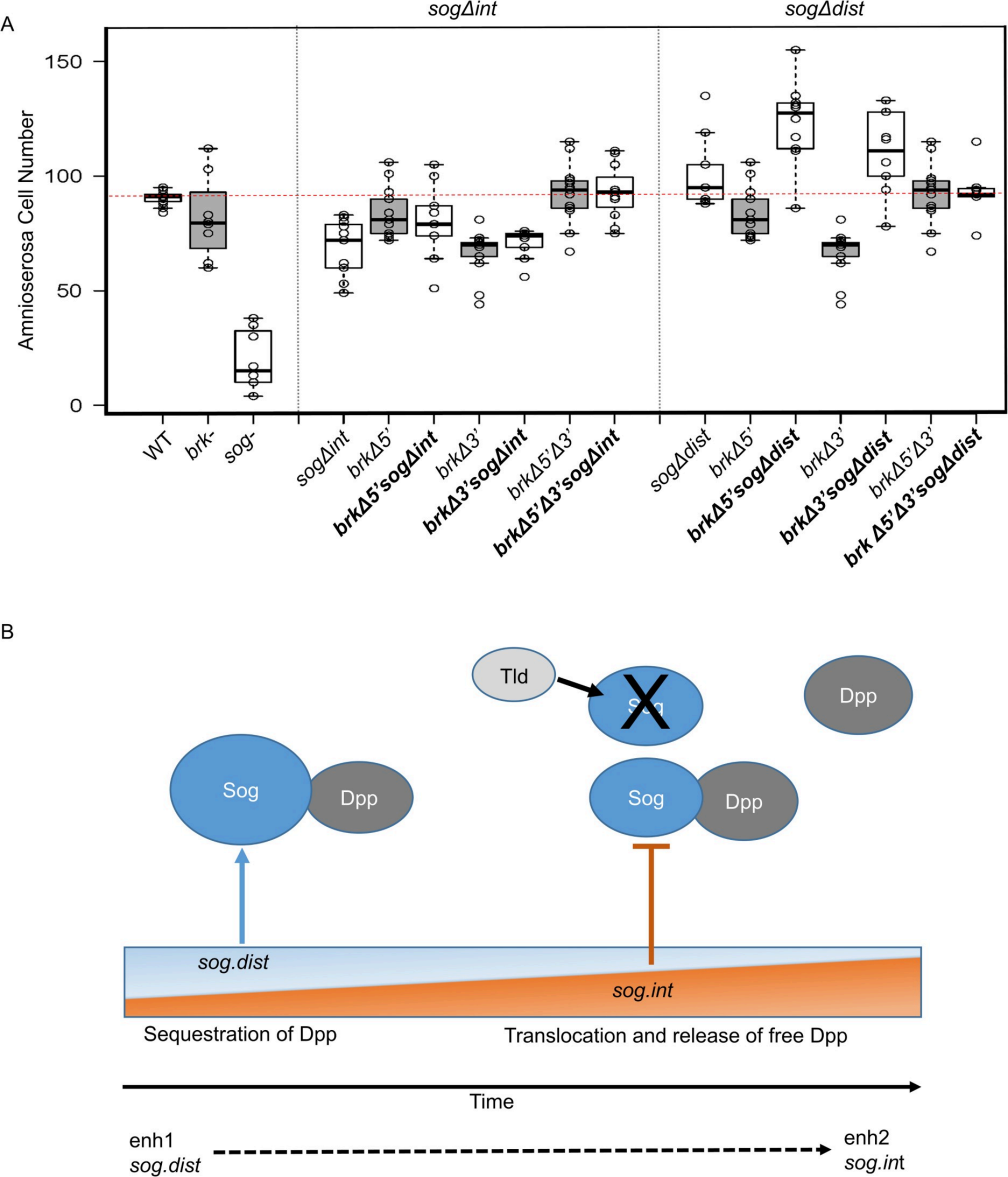
Genetic epistasis between enhancer mutants gives insight into timing of action of *sog* enhancers. (A) Number of amnioserosa cells on the lateral side of the embryo for each of the genotypes. For ease of comparison, results from all *brk* mutant embryos (shaded boxes) are repeated from Fig 1, and the single *sog* mutant embryos are repeated from Fig 6, and placed next to the corresponding *brk* and *sog* double mutants. Amnioserosa cells associated with 8–11 embryos were counted for each genotype. (B) Model of *sog* enhancer action. *sog*.*dist* acts early driving high levels of expression of Sog and leading to an excess of Sog over Dpp resulting in predominantly sequestered ligand. Dominance of enhancer activity switches over time and *sog*.*int* is more influential later, lowering the levels of Sog relative to Dpp, leading to Sog levels which allow for free Dpp and signaling.

To test for genetic interactions between the *brk* and *sog* enhancer mutants, we recombined the three *brk* enhancer mutants into either the *sogΔint* and *sogΔdist* mutant backgrounds. All double and triple mutants were homozygous viable. In addition, we found that all of the *sogΔint* recombinants were epistatic to the *brk* enhancer mutant in terms of amnioserosa cell number, while the *brk* single mutants appeared to be additive with the effect of the *sogΔdist* mutant phenotype (Fig 7A). These genetic interaction results provide insight into the relative timing of action of these enhancers; *sogΔint* likely acts later than either *brk* enhancers, whereas *sogΔdist* is likely coactive with them (see Discussion, Fig 7B).

## Discussion

In this study, we have used a genetic loss-of-function approach to examine the roles for individual coacting enhancers associated with the genes *brk* and *sog* in the early *Drosophila* embryo learning that these enhancers are not functionally redundant but differentially impact gene regulatory networks due to the changing roles of the gene product in time and space. Although the examination of associated gene expression is an obvious first step in understanding their importance, the effect on the broader gene network caused by deletion of individual coacting enhancers has not been closely examined. Our analysis of individual enhancer functions in the context of the gene regulatory network acting in early embryos led to three main findings:

### I. Individual enhancer deletions for these genes are viable and yet provide novel phenotypic insights

First, individual enhancer deletions for *sog* and *brk*, as well as compound embryonic enhancer deletions for *brk* are viable, while the gene mutants are zygotically lethal. Despite the array of phenotypes associated with these genotypes, the fact that they are compatible with viability underscores the robustness of embryonic development in *D*. *melanogaster*. More importantly, however, assay of novel phenotypes associated with these enhancer deletions allowed us to uncover new functions for these genes that had not been previously realized by analysis of gene mutant phenotypes.

In the case of *brk*, the *brk3’* enhancer deletion indicates a role for Brk in specification of amnioserosa and establishing the dorsal border of some ventrally expressed genes. It was the unexpected change in amnioserosa number, specifically in the *brkΔ3’* embryos, that first indicated this altered gene network state (Fig 1J and 1K). Furthermore, addition of NanoString data allowed us to observe that in the *brkΔ3’* mutants a number of genes are upregulated that are minimally affected if at all in the other two mutant lines. Unexpectedly, several of these genes were expressed in the ventral side of the embryo, in the presumptive mesoderm (Fig 3E–3I). This expansion of ventral genes into the lateral neuroectodermal region in *brkΔ3’* embryos highlights a previously unknown role of Brk in repressing ventrally expressed genes. We suggest that this role is likely masked in *brk-* gene mutants by the expansion of BMP signaling that is known to repress many more ventrally expressed genes [42]. In addition, other genes such as *Neu2* and *Su(H)* are repressed in a reverse additive manner, with the most repression associated with *brkΔ5’* and the least repression with *brkΔ5’Δ3’* (S2 Fig). Again, this pattern demonstrates that removal of a single enhancer can lead to a more severe outcome than removal of both early acting *brk* enhancers in these embryos.

**Fig 8:**
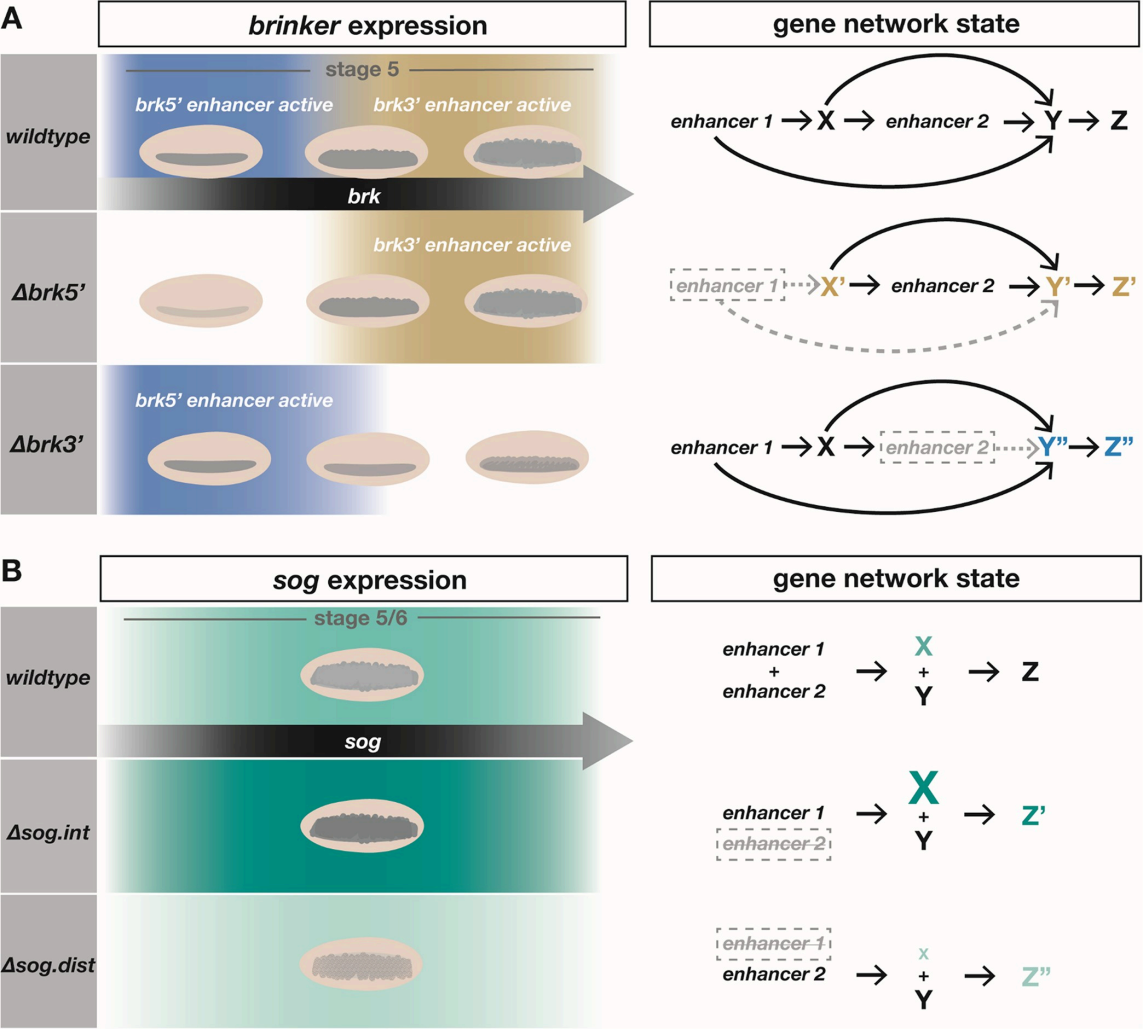
Spatiotemporal and dosage dynamics associated with *brk* and *sog* enhancers differentially affect *Drosophila* embryo gene network. (A) Model of spatiotemporal changes associated with enhancer action in wildtype embryos (top row) compared to *brkΔ5’* (middle row) and *brkΔ3’* (bottom row). Embryo images show spatial expression changes in *brk* during stage 5 driven by the *brk5’* (gold) or *brk3’* (blue) enhancers. Gene models to the right show changes in gene network states. Expression of *brk* through enhancer 1 (i.e. *brk5’*) creates gene network state X which influences the expression of *brk* from both the enhancer 2 (i.e. *brk3’*) as well as gene network state Y and Z downstream. Deletion of enhancer 1 or enhancer 2 lead to alternate gene network states (X’, Y’, and Z’ or Y” and Z”). (B) Model of dosage changes associated with enhancer action showing the change in *sog* expression from moderate in wildtype (top row), to high level in *Δsog*.*int* (middle row), and low level in *Δsog*.*dist* (bottom row). Gene models to the right show changes in the gene network state in each of the genetic backgrounds. The changing levels of X (i.e. *sog*) in the enhancer mutant backgrounds combine with constant levels of other gene network components Y (e.g. *tld*) to give opposite effect on gene network state Z.

We propose that analysis of gene mutants provides only partial insight into gene function, as multiple enhancers often support spatiotemporally-diverse expression and loss-of-function phenotypes may bias analysis toward identification of the role of only the earliest-acting enhancer. Altered expression resulting from individual enhancer deletions does not merely reduce expression of the gene it regulates but alters the regulatory environment within a cell in which expression governed by a later-acting enhancer acts; a novel gene network state is produced leading to less severe but important changes distinct from those observed from gene mutants (Fig 8).

### II. Mutant phenotypes associated with enhancer deletion genotypes uncover complementary roles for pairs of shadow enhancers

Second, we uncovered that individual coacting enhancer deletions can sometimes present opposite phenotypes suggesting these enhancers normally play complementary roles and make nuanced, interdependent contributions to supporting gene expression. For example, the *brk* enhancer mutants allowed us to more closely examine Brk’s role in the early embryo involving refinement of the expression of *dpp* and BMP target genes. Expansion of *dpp*, *tld*, *pnr*, and *zen* patterns is present in the embryos with deletions of the *brk5’* enhancer, but not when the *brk3’* enhancer alone is deleted. This shows that early stage 5 expression of *brk* (predominantly supported by the *brk5’* enhancer) is required to properly regulate the boundaries of canonical *brk* target genes involved in BMP signaling (see Fig 2). Conversely, *brk* expression primarily driven by the *brk3’* is required for the compensatory responses by the broader gene network that refines the final output of BMP signaling (see Fig 4G). A simplified view of this system is that Brk functions twice in a compound feedforward loop (Fig 8A) to support BMP signaling outputs, ultimately affecting tissue specification such as amnioserosa. The initial action is through the *brk5’* enhancer (i.e. enh1; Fig 8A) and the subsequent action is through the *brk3’* enhancer (i.e. enh2; Fig 8A). The action of these enhancers is coordinate (i.e. *brk3’* enhancer action is designed to follow *brk5’* enhancer action), and when one or the other is missing the system breaks down in alternate ways.

Analysis of the *sog* locus gives further credence to the theory that coacting enhancers can support different gene functions. Slight changes in the domain of expression of Sog, as a secreted protein, is unlikely to affect its function, but the levels of Sog in relation to the other components of the GRN, such as Tld and Dpp, is critical (Fig 8B). Despite the lack of obvious difference in their spatiotemporal expression when assayed by reporter constructs, the phenotypes of the *sog*.*int* and *sog*.*dist* enhancer mutants are dramatically different. This functional difference likely relates to slight differences in timing as well as the marked change in levels of expression. Genetic interaction with *brk* enhancers suggest that *sog*.*int* acts after *brk* enhancers, whereas the *sog*.*dist* enhancer acts concurrently with *brk* enhancers (Fig 7A). We infer from these genetic epistasis experiments that *sog*.*dist* acts before *sog*.*int*. Through altering the levels of gene expression over time, these enhancers may differentially affect Sog activity (Fig 7B). We propose that the *sog*.*int* enhancer may function as a damper to downregulate levels of *sog*. Later action by *sog*.*int* may regulate Sog levels to support just enough expression to locally inhibit BMP signaling through sequestration of ligands but to support activation at a distance following cleavage by Tld. These data are consistent with the idea that *sog*.*dist* promotes Sog’s ability to repress Dpp locally whereas *sog*.*int* promotes Sog’s ability to activate Dpp at a distance (Fig 7B), and likely explains why the BMP target gene *Race* exhibits opposite phenotypes in the *sog* enhancer mutant embryos (i.e. expanded versus lost, respectively). Thereby, even enhancers with no obvious difference in temporal expression may exhibit temporally specific, and complementary, actions.

Both of the single deletions, as well as the double deletion, for *sog* have markedly different gene network outputs. Most strikingly, similar to what we described at the *brk* locus, the phenotype associated with the *sog* double enhancer deletion (*sogΔintΔdist)* is not simply an additive effect of the phenotypes exhibited by the single enhancer deletions (*sogΔdist* and *sogΔint*). These results are understandable, however, in the context of how Sog interacts with the other components of BMP signaling [32,43]. When Sog is upregulated (*sogΔint*), more Dpp is sequestered leading to lower activation and a loss of peak level responses, such as *Race*. If, however, levels of Sog are downregulated (*sogΔdist* and *sogΔintΔdist/FM7)*, there is more free Dpp, but less shuttling, leading to a broader area of activation of target genes. Finally, with no Sog present (*sogΔintΔdist*), there is also more free Dpp but no shuttling so that the mid-level targets such as *rho* are expanded but peak levels are not reached to activate genes such as *Race*. The double mutant is lethal but the single enhancer deletions are both viable. Thus, some amount of Sog, even if levels are high or low, is necessary, as the system is able to compensate for aberrant levels but cannot tolerate a complete lack of zygotic Sog.

### III. *brk* and *sog* function to canalize embryonic patterning, in a manner requiring coordination of their respective coacting enhancers

Lastly, our data demonstrate that both *brk* and *sog* contribute to canalization of patterning in the early embryo. Though it has been appreciated that *brk* and *sog* genes work redundantly to regulate patterning in the early embryo to support refinement of expression of genes such as *zen* and *dpp [11]*, a role for Brk in the regulation of *sog* had not been previously described. In the *brk* mutant, the increase in *sog* levels (Fig 2F) as well as concomitant decrease in *tsg* (S4A Fig), the cofactor which facilitates long range translocation of Dpp, could lead to more Dpp ligand remaining sequestered by Sog in the lateral parts of the embryo and not being transported to the dorsal midline. Higher local Sog levels, produced in this way in *brk* mutant genotypes, may serve as a compensatory response to the higher Dpp levels that result from loss of *brk*. Thereby, as long as *sog* is present, the system appears able to adjust and compensate for lack of *brk*. Brk has not been linked previously to canalization of patterning in early embryos, though it has been shown to support dorsal closure at later stages when embryos are subjected to thermal stress [44].

Canalization of dorsoventral axis patterning of the *Drosophila* embryo was characterized in a previous study, which used amnioserosa cell number as an indicator [45]. The number of amnioserosa cells was found to be tightly controlled in *Drosophila melanogaster*, with little individual variation present except when two members of an identified canalization circuit, *egr* and *Cv-2*, are both removed. Both *egr* and *Cv-2* require early *zen* for expression (S5D Fig) [45]. In the *brk* enhancer mutants, we also detected large variation in amnioserosa cell number specification in both the *brkΔ5’* and *brkΔ5’Δ3’* embryos, suggesting the canalization circuit is regulated by Brk. This idea is supported by ChIP-seq binding data, which show differential binding of Brk to the pathway components at the two time points: binding to *zen* early, *egr* late, and *Cv-2* early and late (S5E–S5G Fig) [28]. In addition, the three genes are all differentially expressed in the *brk* enhancer mutants: the loss of expression of *brk* early (*brkΔ5’* and *brkΔ5’Δ3’*) leads to a loss of *egr* expression, especially in early stage 5, but an expansion of *Cv-2* expression; while a loss of late *brk* expression (*brkΔ3’*) gives expanded *egr* expression, but no visible change in *Cv-2* (S5A–S5C Fig). This lack of change in *Cv-2* may explain why *brkΔ3’* embryos can still regulate the number of amnioserosa cells, possible when one branch of the canalization circuit remains intact; while the other two mutants do not properly regulate number of cells, as both branches are aberrant.

Previous studies of coacting/shadow enhancers have found that complete redundancy in spatiotemporal expression driven by these elements is rare, or possibly non-existent [6]. Yet the similar patterns of expression were thought to have evolved for evolutionary robustness of the gene in the face of genetic or environmental perturbations. Here we have used genome editing to manipulate individual enhancers in co-acting pairs to examine effects on target genes. The changes in the gene network state upon removal of a single enhancer demonstrate that the slight differences in timing, levels, and expression domain supported by these enhancers can have much larger effects on the gene network as a whole. By working in complementary ways, these coacting enhancers’ native functions are interdependent and serve distinct roles in the context of dynamic gene regulatory networks, pivotal for robustness and canalization of patterning.

## Materials & methods

### Fly stocks and genetic crosses

All flies were reared at 23°C on standard fly media. *yw* was used as a wildtype control unless otherwise noted. Fly stocks used in this study are: *brkm68/FM7c ftz-lacZ* and *brkM68 sogYS06 / FM7c ftz-lacZ* [11], s*ogY506/FM7c ftz-lacZ* [46], *brk 3’-evep-lacZ* and *brk5’-evep-lacZ* [10], sog.int-evep-lacZ [47], sog.dist-evep-lacZ [12], and His2Av–RFP fusion [Bloomington *Drosophila* Stock Center (BDSC) #23650].

For CRISPR-Cas9 deletions within the genome, gRNA constructs were created by modifying the pCFD4 plasmid (Addgene Plasmid #49411, [48]) to target PAM sequences flanking the region to be deleted. flyCRISPR Optimal Target Finder (http://flycrispr.molbio.wisc.edu/tools) was used to identify the PAM sequences with no predicted off target hits. The gRNA plasmids were then injected into either y2 cho2 v1 P{nos-phiC31\int.NLS}X; attP2 (III) (NIG-Fly #TBX-0003) or y1 v1 P{nos-phiC31\int.NLS}X; attP40 (II) (NIG-Fly #TBX-0002). Stable gRNA transgenic lines were created and then crossed to a Cas9 expressing line (y2 cho2 v1; Sp/CyO, P{nos-Cas9, y+, v+}2A, NIG-Fly #Cas-0004). Individuals from the next generation were screened by PCR for the deletions. Alternatively, for the *sogΔint* and *sogΔdist*, a homologous recombination cassette (HRC) was created by adding 1kb homology arms to the plasmid pDsRed-attP (Addgene #51019, [49]) and this HRC was injected into embryos heterozygous for the gRNA and Cas9 transgenes [49]. Progeny of the injected embryos were screened for RFP expression in the eyes. The RFP cassette was then removed by crossing these flies with a Cre expressing line (FM6:sna[Sco]/CyO, P{w[+mC] = Crew} (recombined from BDSC 1093 and 784), leaving only an attP site in the integration position. *brkΔ5’Δ3’* was made by crossing *brk*Δ5’;P{nos-Cas9, y+, v+}2A flies to FM6: brk3’gRNA flies and screening by PCR for the double deletions. Similarly, *sogΔintΔdist* was created by crossing *sogΔint;*P{nos-Cas9, y+, v+}2A flies to FM6: sog.dist’gRNA flies and injecting the heterozygous embryos with the *sogΔdist* homologous recombination cassette (HRC). Lines were screened for RFP in the eyes and the RFP cassette was subsequently removed in the same way as for the single mutants. All lines represent isogenic backgrounds differing only in the gRNA line used to create the specific deletion.

### In situ hybridizations and antibody stainings

Embryos were fixed and stained following standard protocols. Antisense RNA probes labeled with digoxigenin, biotin, or FITC-UTP were used to detect reporter or in vivo gene expression as described previously [50]. Probes for *sog*, *aos*, *zen*, *sna*, and *rho* were transcribed from cDNA subcloned into pGEM-T vector or full-length cDNA as for *brk* (RE18244, DGRC). For fluorescent *in situ* hybridization (FISH), probes were detected using Sheep anti-digoxigenin (Life Technologies PA185378), Mouse anti-Bio (Invitrogen 03–3700), and Rabbit anti-FITC (Invitrogen A889). Fluorescently labelled secondary antibodies were all from ThermoFisher (used at 1:400). Enzymatic detection was performed using digoxigenin labelled probes followed by detection with Anti-Digoxigenin-AP antibody (Sigma, 11093274910) and standard AP staining using NBT/BCIP. Other antibodies used in this study were Hnt (1:20; DSHB 1G9), pSMAD1/5 (1:30; Cell Signaling Technology, 9516), and dpErk (1:200; Sigma, M9692).

Specific staging of embryo images is indicated in the associated figure legend. For early and late stage 5, the shape and length of the nucleus as well as the membrane front of the cellularizing cells was used to define either early or late stage 5. Early stage 5 (nuclear cycle (nc)14 A and B) included all embryos in nc14 (determined by nuclei number, see [22]) that are not fully cellularized; while late stage 5 (nc14 C and D) included embryos that are fully cellularized with the membrane front fully encapsulating the nucleus, but before gastrulation. Early stage 6 embryos were identified by the small indentation at the anterior of the embryo which begins the formation of the cephalic furrow. Embryos of each genotype were imaged in bright field through early embryogenesis. Although the time from the start of the movement of the cellularizing membrane front to posterior midgut invagination varied by ±5 min from the wildtype average of 33 min in two of the mutants (i.e. *brkΔ5’* and the *brkΔ5’Δ3’*), there were no obvious defects in cellularization seen in any of the mutants. Using nuclear shape and size combined with percent cellularization to characterize developmental stage was judged to be an adequate method to synchronously stage embryos that is independent of the genotype.

The following primers were used to generate riboprobes:

dppi-f ccagaactagaaaaccggaagc

dppi-t7-r gaaatTAATACGACTCACTATAgggCGCCTGTGCTAAAGACCCTG

stumps-f TGGCCCAGAACATCGTCAGTTT

stumps-r-t7 gaaatTAATACGACTCACTATAgggATGAGACTTCACCTGCTCCTGGAT

tld B-f ATGTGGATGAGTGTTCAAT

tld B-r-T7 gaaatTAATACGACTCACTATAgggTCCCTTCGCTGGACCTCTCAT

Race-f ATGAGACTGTTTCTGCTAGCCCTGC

Race-T7-r GAAAATTAATACGACTCACTATAGGGACGCAAGCAGAAGGCACAGATA

netA–f ATGATCCGTGGAATCTTGCTCCTGC

netA-T7-r AATTTAATACGACTCACTATAGGGCTTTGCACTCATTGGCTTCCTTGGC

pnr-f ATCTCAAACCCTCGCTCAGC

pnr-T7-r aagtaatacgactcactatagggagaCGAGGTGGCCATCAGTTTGG

sog ex1-f TCAGGTTCAGTCGCTCTTGA

sog ex1-T7-r AATTTAATACGACTCACTATAGGGGTGTCGGACTCCTCGAACAT

### Quantitative analysis of stainings

Embryos were sectioned along the anterior-posterior axis manually using a razor blade, and cylindrical mid-embryo sections were mounted on the cut side allowing for imaging of the axial plane. Percentage of embryo circumference for *dpp* and *rho* was measured using ImageJ. First, an ellipse was fit to the outside of the embryo image, and the perimeter of this ellipse was measured; then, an arc was manually drawn along this ellipse matching the extent of fluorescent signal for each gene measured. The length of the arc was measured, and a ratio of arc length to full circumference was determined. The width of *zen* at stage 6 was measured using chopped sections that were co-stained with DAPI to mark each of the nuclei. The cells expressing *zen* were counted manually. The amnioserosa cell number was counted using stage 10–13 stage embryos stained with Hnt antibody. Nuclei on the lateral side of the embryo only were counted manually in ImageJ by planing through the z stacks and using the Cell Counter plug-in. Box plots were created using BoxPlotR (http://shiny.chemgrid.org/boxplotr/).

The width of the pMad gradient was measured as described [45] with the exception that the area selected for analysis was 120 μm along the D/V axis by 200 μm along the anterior-posterior axis, with the top of the selection aligned to the developing cephalic furrow. The embryos from different backgrounds were all collected, fixed, and stained simultaneously. Imaging was done on a Zeiss LSM800 microscope with a 20X objective. Imaging for all backgrounds was done within 2 days using the same settings for all collections. The width of the gradient was determined as described [51]. In short, it was assumed that the measured intensity is proportional to the concentration plus background noise. To calculate the scaled concentration curves that minimize *χ*^*2*^ [51], we used a custom R script. We set the maximum of the mean pMad concentration of the WT embryos to one and the minimum of the mean concentration to zero and normalized the mutant data to the WT data similarly as described [51]. The scaled curves were cut at 40% of the wild type amplitude using a custom Python script to extract the intersection points and to calculate the width. The measured widths were plotted in R using the boxplot function.

Heatmap representation of images for pMad and *stumps* were made in ImageJ. Z-stack projections were created using the sum slices function and then the heat map was applied using the LUT function.

### Embryonic viability assay

Grids were drawn on standard 10 cm apple juice collection plates, dividing each into 160 quadrants. One embryo was transferred from a two hour collection into each of the quadrants of the grid. The plates were aged at 25°C in a humidified chamber for 30 hours, and the number of hatched and unhatched embryos was manually counted. This was repeated for a minimum of three times on different days for each genotype.

### NanoString analysis

NanoString was run as described [22]. H2A-RFP live DNA marker (BDSC #23650) was crossed into all of the *brk* mutant lines and nuclear morphology was observed on a Zeiss LSM800 microscope. Embryos were collected at nc14C for each genotype. This specific stage was determined to be when the nuclei were elongated but not yet disorganized, and the membrane front (visualized in brightfield) fully encapsulates the nuclei [22]. Once extracted from individually staged embryos, total RNA was hybridized with NanoString probes at 65°C for 18 hours and then loaded onto the NanoString nCounter instrument for automated imaging and barcode counting. To normalize between embryos and runs, l μl of Affymetrix GeneChip Poly-A RNA Control was spiked into Trizol with each embryo at a dilution of 1:10000 before RNA extraction. A total of 6 (*WT*, *brkΔ5’Δ3’*) or 5 (*brkΔ5’*, *brkΔ3’*) individual embryos were analyzed for each genotype.

### Primers used and quality control of CRISPR-Cas9 generated deletions

Individual deletion lines for *brkΔ5’ and brkΔ3’* were verified by crossing deletions made with different gRNA lines in trans to check for offsite effects. For *brkΔ5’Δ3’*, two independent mutant lines made using the same gRNA pair was crossed in the same manner. No differences were seen between the single mutant embryos and the trans-heterozygous embryos in the expression of *brk*, *dpp*, *Race*, or *zen* so all analyses were done using a single mutant line for each deletion.

As an additional control, transheterozygous embryos for enhancer deletions and respective gene mutant alleles were analyzed to ensure that the phenotypes ascribed to enhancer deletions relate to changes in *brk* or *sog* gene expression, specifically. For *brk* analysis, males of the genotype YW, *brkΔ5’*, *brkΔ3’*, or *brkΔ5’Δ3’* were crossed to females of genotype *brkM68/FM7 ftz-lacZ*. To identify trans-heterozygous embryos (i.e. females of genotype WT/*brkM68*, *brkΔ5’*/*brkM68*, *brkΔ3’*/*brkM68*, or *brkΔ5’Δ3’*/*brkM68*) and assay for changes in amnioserosa, embryos were stained with a combination of antibodies: anti-βGal to detect the balancer, anti-Sxl to identify female embryos, and anti-Hnt to detect amnioserosa. Similar trends were seen as for homozygous enhancer mutants (see S6 Fig). These trans-heterozygous lines were also assayed by FISH for *tld* expression and were found to be consistent with the *tld* expression pattern of the homozygous *brk* mutants (S6 Fig compare to Fig 2C). A similar control for the *sog* mutants was not possible as *sog* is dosage dependent. We have included the phenotypes of the *sogΔintΔdist* heterozygous embryos for each of the analyses in the main panel to show that the phenotypes for the mutations are consistent with the *sog-* heterozygous embryos. Thus, indicating that the changes in expression are due to changes, specifically, in *sog* expression and not due to second site mutations.

Primers to make gRNA plasmids:

Set 1 (used to generation deletions at indicated loci and for phenotypic assays presented):

3’grna-f attttaacttgctatttctagctctaaaacCGCCTCGGCCGGCGTCGCTGCgacgttaaattgaaaataggtc

3’grna-r attttaacttgctatttctagctctaaaacCGGGGTGGAAAAGCGACCCGCgacgttaaattgaaaataggtc

5’grna-f tatataggaaagatatccgggtgaacttcGTACCTGTTCGATCCTTCATgttttagagctagaaatagcaag

5’grna-r attttaacttgctatttctagctctaaaacTTGGGCTTTCGTTGCACAACgacgttaaattgaaaataggtc

Sog.int grna-f tatataggaaagatatccgggtgaacttcGTCAAAATCTTTAGTTAAAGgttttagagctagaaatagcaag

Sog.int grna-r: attttaacttgctatttctagctctaaaacTAGATCCCGGGATTTGTGCCgacgttaaattgaaaataggtc

Sog.distgrna-f:tatataggaaagatatccgggtgaacttcGATGATAGGTGGACTCTTGATgttttagagctagaaatagcaag

Sog.distgrna-r:attttaacttgctatttctagctctaaaacAGCGAATACGTGGAATTCCTCgacgttaaattgaaaataggtc

Set 2 (second independent generation of deletions used to test for 2nd site mutations):

3’grna2-f tatataggaaagatatccgggtgaacttcGTTCCAAAACTTTAATCTGTTgttttagagctagaaatagcaag

3’grna2-r attttaacttgctatttctagctctaaaac GCAATCATTCTTCAATTCATCgacgttaaattgaaaataggtc

5’grna2-f tatataggaaagatatccgggtgaacttcGACCCGATGAAGGATCGAACgttttagagctagaaatagcaag

5’grna2-r attttaacttgctatttctagctctaaaacGCTTTCGTTGCACAACTTTAgacgttaaattgaaaataggtc

HDR templates:

Sog.int LA-DSR-f gtacgtgaattccgaaactcgcgtgtgttatcta

Sog.int LA-DSR-r ctagcggcggccgctaactaaagattttgactagt

Sog.int RA-DSR-f gtacgtggcgcgccatcccgggatttgtgcc

Sog.int RA-DSR-r ctagcgctgcaggcggcagacagttgaataaa

Sog.dist LA-DSR-f gtacgtcccgggtccatccccacaccatttat

Sog.dist LA-DSR-r ctagcggcggccgcgaatacgtggaattcctttcg

Sog.dist RA-DSR-f gtacgtactagtaagagtccacctatcatcccagt

Sog.dist RA-DSR-r ctagcgggcgcgccagatgcgccagaagtacg

CRISPR-Cas9 Deletions:

Deleted base pairs capitalized. Indel/added sequence shown in bold italics.

*brkΔ5*’:

tggtattaaaactgaaaatcaatctaaaaatcaaccattgataacattttattgaatcaaaccaaaagccaaattgattcctgaatccaaaagacccgatgaaggatcgaacaggtactacgatgatattggtcgGAAAATACCTGCGCATCCTGGTGGTTTATGGTGCGGCCGTAAATGCAAGCCAAGTTCTTTACGGCTTCTCTGGCACAAACCCTAAATGTGGATTACGCTAATATTGCCCCCCCTAATAAAAACGGTCGTTGTCCAGGGCCGAATATTGCGTCTGATTGGTTTTTCCCACGATTACAATTAGCCGGACGGACACAAACTGACCTGAGCTGACCCGCAAAAAGACACGGTTGTCCGGCAGTCGGAACTGAAGGAAACTAAAGGAAACTGAGGGCAGGTCAGCGCTATGGATTGTGCACTAAGTTGCTTAATCCGACGGGAAATCCAAAACACAACCCGAGCCCGATCCTTCGCTCCTTCGATTTAAGCCAAAGTTAGAGGCACAGGCACACATGTGTGTTTGGTTTGAACGGGAAAGCCCCATTTTAAAGCTGGCCAACCAACGGCAACACATGTTCATGTTAGGACCGATACAGGTTGACATTCCCTGGAAGGATGCACCTCTGGGAGATTCCCACAACCGGCAGCAGGTCATGTCCAACCGATCGTTGCGGGAGCCACTTGTCCCGAAAAAATCCAAAGAAACTATCAAGTGGCGTTTAGGGAAACTCAAAACTTTCCAACCACACCATATCTTTCTAACGCCACACAATAAACTGGTATGATCACTGTTAAGATCAAAATGGGCTAAATAAAGCGATCATGAATCATTTACTATAAACTGAAGAGTTTTCTATTCTTTAATATAAAAGAAAAGATGAGTAACACCCTACAAAAATTTTAGGACTACAGATCTACCCACCGATGATGATCAACCCTTCCCAAAAAAAAGTCACAGCTTGTATTTCGTAAAGATTTCAGATTTCTTAAGACACGATCACCAACTGATCTATGGACTTCTTAAACCATTGGGCTTTCGTTGCACAACTTTAGGGATTTGTTTTTTTTTTTTTTTTTTTGATTCCACATCCGATTTCTTCTTCGAAGCACTTCGATCTTTTCTCacatcagtttgatcgacaatcgacgatcgatggctgtgtgaaaacaagtttcgtaactatgtaaattaacctcca

*brkΔ3’*:

cgagacgggcagaaagaacgagaggaatggagatagaggg***GGAGAGCAGAGAGCA***AGCAGGAGCCAAGAGGCAAAGCCAAAAGAAATGCGTTTCTTTTATTTTTGACGCGTCTTAGATTCTTCCTCTGCCGCTTCTTCTCTCGTTTTTTTCTTTCACTTTTTTGTTTTTTTTTTTTTTGTTTTTGATTTCTTGGAATCGCATCTACTTTGAACCTTCCACGTTCCGACGGAAGACTCAGAGACTCGACTGCATTGTATATTTGAATTTCATTTCAATTTAAATACGATTTTCTATGAAAATGCACGCACCACAAATACGTGCAATCGTTATCCAAACCATCTACCCCTCCCCTCCTCCCCTTTATCACCATGCTCTTTGCCTCCGCCGTCCTCTGCTTTTTTTTTGTTTTTTACCCCATACATGTACATAATACGATCCAAGTTTCATGAAAAAAACCGGAGAAACAAATTGATCATGTTTTGAATGCATTTCTGTTTGATTTCAATTTCGATTTGTGTACTCCATAAATGCGAAAGAATCTTGGATTGGAGTTGGGGGGATAAAATGGATGGATATATGGGGTGGGGTAAATCAGAATTATACGGCATATGCGGATCTAATCTAATAATATACAATATTACTGAGTCGCATACCCTAGAAATCGCACATTGATTAGCACAACCTGTCCAAATTATCCGAGCAAAATCCCCCCAAATCACGCAACACTCTAAACTGATCCGAAAGATCCTAACAGTTGAAAATCCACCTAGTCGAGCTAAAACCAAATTGTCAGAGGATTGCCTTGAATGGCCCACTTAAGCCAGGCGTGCGGCTGAAAGGAGCCGAGGATTCCTTGCCGAATCCGAATCGGAATCCTGTGCCAAGGACAGCATGCAACGCACCGATGCGAGTGCCGAATGACCTTTGGGAAAATACGTGTTGATCGACATCCATCGGCAATTGTTTGAGATCTTCGGAAATCTCTCACCTTTCGAATGGGAAATTCCTTAGGATCATCGAGCTAGCTGGTCAGAGAAGGCCGTTCATAAGCATGCAAATAAAGATTTCACACCTCGGCCAGCGCACTAGGGTGCATGGTGCAGCATAGGGCTCTCAGCCAAAGGACACGAAACGAAATGCATGGAGATCGGGACACCTGGGAAATACCCAACCTGCCCGAAACATATGGACGCACAACGCACAGGAGGGATCTCTCGCTCTGAATGGAAAGCAGAAACTCTCTATAGGCACTAGCTATCCATCCAAGTGGTTCCCGAAAAAAAAAACCCTCACAATATAATTCGTAAATCCCAGCCGCAGGTATTTAGTTCCGCCTTTGTCCATCTTTGGAATTCCCAATGCTATTATTGCACCTAAGCCACTGGAGTGGGAGGTTTTGCAGTGTGTGTGCGCGTTGCAAGTTGCTGCCGGTGAGCAACACATCGAATTCGTCCCATCTCTGTCTGGCCGCGACTCGTCATCTCTCTCTCTCTCTCTCTCTCTCTGATCTCTCTGTCCCGCTCTGGTTCGTGCTCTTGCTGTCGCCGGCGCCGCAGGTCAACAGCGATGTGTGGCATTTTCTAACAGTTGCTGCTGCTGTCGCAGGTTGCAGGTTGCATGTTTTGTTGCAGTTGCTGCAGCTTATATGACGCTTACATTTGTTATATGTATCTGCCACTGGGGGTTTTCCGTCAATATTGCAGGTTGCATAGCTTATGCTGCAGCTTCTATTGATGACTAATTTATTGTTGCTTGCTTGCTTGAGCAAGTTGCTTTAGGTTTACATTAAAACGGCACATTTCTTGGCATCAGTTGCACTCTCTGTTGGTATAATTGTTGCTGTTTCTAGTATCAAGTGTACTAATGTTGCAGGTGCTGCGCATGCATATGTTGCAAGTCTGTTGTTTATTGTAATGTACCCATAAAACTGCATATATTTATACATAGGTGTTTAGATTTCTACTATTATCGCTGAAAACTTGTTTTTCTATTCAATCTTGCTCATAAAATGGTTCAAATATATATAGATTTGCTGCACTCCCGACTTAATTCTTCCAAAATGGCTGTTAAATTTCAAACTATTGCCCATGTTGTTTTCGTTTAAATCGCTTATGGTTTTTCGGATGTTGTTTTTCGTTCAATCGCTGGCTGATTTTCTTGCTCTCCCGAAAATCCACACGCACATTTTCATTGTCGACAAATTTTCAGACGGCAGGACAAAGAATTTTTCATTCGTTTGTTTTCCACGCACTCGGTCTGAATGGCCAAAAAGGAAAATGTGACGAGCTAGAGGGAAATAGGGCACATTTTTGGGGGCAAGCGAGATGACCAAAAAAGACCTGCGATCCGTTCCGATCCGCTCCGGGTCGCTTTTCCACCCCgggggcattgttttcagaccgagagtatatagtagaatctcctattatatcttctattcctatttgtatttgtatttgta

*brk3’* in *brkΔ5’Δ3’*:

gaagcgaatgccaatgcacaaggagctaaacgagtgcgagcgagacgggcagaaagaacgagaggaatggagatagagggAGCAGGAGCCAAGAGGCAAAGCCAAAAGAAATGCGTTTCTTTTATTTTTGACGCGTCTTAGATTCTTCCTCTGCCGCTTCTTCTCTCGTTTTTTTCTTTCACTTTTTTGTTTTTTTTTTTTTTGTTTTTGATTTCTTGGAATCGCATCTACTTTGAACCTTCCACGTTCCGACGGAAGACTCAGAGACTCGACTGCATTGTATATTTGAATTTCATTTCAATTTAAATACGATTTTCTATGAAAATGCACGCACCACAAATACGTGCAATCGTTATCCAAACCATCTACCCCTCCCCTCCTCCCCTTTATCACCATGCTCTTTGCCTCCGCCGTCCTCTGCTTTTTTTTTGTTTTTTACCCCATACATGTACATAATACGATCCAAGTTTCATGAAAAAAACCGGAGAAACAAATTGATCATGTTTTGAATGCATTTCTGTTTGATTTCAATTTCGATTTGTGTACTCCATAAATGCGAAAGAATCTTGGATTGGAGTTGGGGGGATAAAATGGATGGATATATGGGGTGGGGTAAATCAGAATTATACGGCATATGCGGATCTAATCTAATAATATACAATATTACTGAGTCGCATACCCTAGAAATCGCACATTGATTAGCACAACCTGTCCAAATTATCCGAGCAAAATCCCCCCAAATCACGCAACACTCTAAACTGATCCGAAAGATCCTAACAGTTGAAAATCCACCTAGTCGAGCTAAAACCAAATTGTCAGAGGATTGCCTTGAATGGCCCACTTAAGCCAGGCGTGCGGCTGAAAGGAGCCGAGGATTCCTTGCCGAATCCGAATCGGAATCCTGTGCCAAGGACAGCATGCAACGCACCGATGCGAGTGCCGAATGACCTTTGGGAAAATACGTGTTGATCGACATCCATCGGCAATTGTTTGAGATCTTCGGAAATCTCTCACCTTTCGAATGGGAAATTCCTTAGGATCATCGAGCTAGCTGGTCAGAGAAGGCCGTTCATAAGCATGCAAATAAAGATTTCACACCTCGGCCAGCGCACTAGGGTGCATGGTGCAGCATAGGGCTCTCAGCCAAAGGACACGAAACGAAATGCATGGAGATCGGGACACCTGGGAAATACCCAACCTGCCCGAAACATATGGACGCACAACGCACAGGAGGGATCTCTCGCTCTGAATGGAAAGCAGAAACTCTCTATAGGCACTAGCTATCCATCCAAGTGGTTCCCGAAAAAAAAAACCCTCACAATATAATTCGTAAATCCCAGCCGCAGGTATTTAGTTCCGCCTTTGTCCATCTTTGGAATTCCCAATGCTATTATTGCACCTAAGCCACTGGAGTGGGAGGTTTTGCAGTGTGTGTGCGCGTTGCAAGTTGCTGCCGGTGAGCAACACATCGAATTCGTCCCATCTCTGTCTGGCCGCGACTCGTCATCTCTCTCTCTCTCTCTCTCTCTCTGATCTCTCTGTCCCGCTCTGGTTCGTGCTCTTGCTGTCGCCGGCGCCGCAGGTCAACAGCGATGTGTGGCATTTTCTAACAGTTGCTGCTGCTGTCGCAGGTTGCAGGTTGCATGTTTTGTTGCAGTTGCTGCAGCTTATATGACGCTTACATTTGTTATATGTATCTGCCACTGGGGGTTTTCCGTCAATATTGCAGGTTGCATAGCTTATGCTGCAGCTTCTATTGATGACTAATTTATTGTTGCTTGCTTGCTTGAGCAAGTTGCTTTAGGTTTACATTAAAACGGCACATTTCTTGGCATCAGTTGCACTCTCTGTTGGTATAATTGTTGCTGTTTCTAGTATCAAGTGTACTAATGTTGCAGGTGCTGCGCATGCATATGTTGCAAGTCTGTTGTTTATTGTAATGTACCCATAAAACTGCATATATTTATACATAGGTGTTTAGATTTCTACTATTATCGCTGAAAACTTGTTTTTCTATTCAATCTTGCTCATAAAATGGTTCAAATATATATAGATTTGCTGCACTCCCGACTTAATTCTTCCAAAATGGCTGTTAAATTTCAAACTATTGCCCATGTTGTTTTCGTTTAAATCGCTTATGGTTTTTCGGATGTTGTTTTTCGTTCAATCGCTGGCTGATTTTCTTGCTCTCCCGAAAATCCACACGCACATTTTCATTGTCGACAAATTTTCAGACGGCAGGACAAAGAATTTTTCATTCGTTTGTTTTCCACGCACTCGGTCTGAATGGCCAAAAAGGAAAATGTGACGAGCTAGAGGGAAATAGGGCACATTTTTGGGGGCAAGCGAGATGACCAAAAAAGACCTGCGATCCGTTCCGATCCGCTCCGGGTCGCTTTTCCACCCCggcattgttttcagaccgagagtatatagtagaatctcctattatatcttctattcctatttgta

*sogΔint*:

tcaatcgcatatccttaaagttcgaactagtcaaaatctttagtta***GCGGCCGCGGACATATGCACACCTGCGATCATAACTTCGTATAATGTATGCTATACGAAGTTATAGAAGAGCACTAGTAAAGATCTCCATGCATAAGGCGCGCC***AAGGGGTATTAACTGTTGCCTGTTGCTTTCGACATTTCTACCTCCGCTGCGATTGCATAAGTTGCCAATGCCATTGCGCATACGCCGTGTCGTCTATATGGCTATATGGCTATATGGCTGTATGGTGCGGGGAAATCCCCGTAATCGCAGGTAGAATTCCAGCCGGTGCCGAGGCGGGACCTGCTCGCACCTCTAATCCCGCCAGGGTTTTCGGGACATGGGATATTCCCGACGGCACAGCATAGCACTCCGTTTTCTTTTTTTTTTTTTATTATTATTGTGTCCAGTTTTAATCCGGAAAGCGGGAATTCCCTTCCGCTCGCTGCCTGCACTGCGCTGCGCAGACGCATCGGCGTCCGTAAGCCGCTTACCAAAAAGATACGGGTATACCCAAATGGATGCCTGCCCATGTATATAGACCATTGGGTGGTATGGACCATGGACCATAAAGCGGCACCCAATTGCAATTTGTTGCAACTCACACGCTTGATTAGCTGCATTTCCTGTTTGCAAACCCCCCTCCCAATACTCCCTACACTCATATATTATATATGCATATAAATGTATGTGTATATTGCATACTATTATGTTCCCGAGTTGCAGTTTTTTTTTCCTTTTTTTTCCACTATTTCTTTCTTGGTTCGCTGCCGTTGTTGCATTTTCAATTAAAAACAGATTGCGGTTTACGCTGCAGCGACTTTGAAACGTTACTCGGCTTACACACAAAATGCAGCGCGATAGTAATGATGATTAACAGGTTATGTTAGGGCTTAGTTGATACGCCGTAGatcccgggatttgtgccggaaccggaacgaatatcgaa

*sogΔdist*:

caatcgattaaaattacctctccagttgcacagatcaacggggtagtccacattccattccatcgaaaggaattccacgtattcgc***GGCCGCGGACATATGCACACCTGCGATCGTAGTGCCCCAACTGGGGTAACCTTTGAGTTCTCTCAGTTGGGGGCGTAGATAACTTCGTATAATGTATGCTATACGAAGTTATAGAAGAGCACTAGT***GGGTAGTCCACATTCCATTCCATCGAAAGGAATTCCACGTATTCGCTGGGCGTTTCGGGGGCGGACTGGGCATATGGGCGTGGTGGGAGGGGGTGGGCGTCACGGGGCGTGGCCACACGTGAAACGGCTAATTTGTTGATGCAAATGCGGGTGTGGCCTGTTCTCGGATCGGAGTCCCCAGCACGCGCCCACAAATAAATAAATGGCTGGAGATGGAAATGGAAACCGATGACGGGGAATAGGGAATGACGGCTGGGTAGCGGGGGAGACGGGGATGGGAACTGGGGGGCTGGGGTCTTGGACCGAGATCGCAGCCAATGGCGACAATTGGCATTCACCGTCGCAATTAATCATCATCACCGTCCGATTGACAATCGGGCCACAAATAAATCAAAATCAAAGGCATAAGAAAGCGAATGCAGCGTCGGCATACAACTGCACTGAAAGAAAAATATGTATGAAGCTTCTGGTCAAGCAAACCGCAAGAAGCGCATTTAAGCTCAAGTATGCTCAAGTATTTGTTGTGTAACTTTAGATGATTCAAAATCGCTTTCTTATGTCTTTTTTGACTGCACTTGCCCTGAATTTCGTCGAGTGTAGTTCCCAATCATTGCAACAATTCATCAAGTATGCAAACACACGCGGCCGAATCAATCAATGGCGAGTAGTAGGAGTGCATGGAGGATGAGAAGGAGGAGAAGTTGGTTGAGAGGTCATCGTTGCGATTCTGCGATTCAGCAGTTCCACAGAAGGTGTCGTAATCCTGGACGCAAGGGTGCACGGACCAACTGACAGGGGCAAGTGCGTCCTGTGCCACCAGATGACGCACGATGCGGCCGGAAAAACCCAAAATCAAAAACCGAAAACCGAAAACCTGGTCAGAGTTTCCGAAAACCAAAGAGCCAACATCGAATGCGGCACAATAACCCGATTGTCTGCGAATACCCACGATGATCTAGAATCGCACGGAGAGCACTCTCACGCATCCGTGGCCATATGGGTGCGGCCAAATCGGAAATTCCCAGGACAGGTAGAATGCATTGGATATACGGGTATACGGATTGGAATTGGGATTGGGATTGGGACTAGCACCAGGTTGCAACGCCCGCCAAGAAGCCAATTTAAATAAGCAGCATAAACAAAAGCGACAGCGTTTTATGATCCCCGCTCCTTATCCTTGCACAAGGATATCGCCATGGCCACGCAGGTAGGAATAGCAGATATGGCGGCAATGATGCGCCAACCGCACTGCTTCGTCCTGGTCCTGGTCGGATGGGCTTTTCCCACGCAACCGCGACCTTATCTGCGCCCCTTTTATGAGGCTGCATCTGTTTTCGCACCTCGATGCCGTTGGCATTATAGCCACATGTGTATGGTGGGAATTTCCGATCGACCAGCCTACCTGTTCCGCTGAAACCCGGGAATCTGTCCATCCTGAGCTTCCACACACACACACACACACACACAGGTCAGTCGGCATCAATTGGCTGCCATAAACATATAACAATCAATATTGAATCCTTTATCGTAGAATTTGTTGTATATGCCCATTGCAGTCCTTCGATTAAATGATGGCTAAAATGAATAAAATGAGTTGCTAGGTTCGAAGTAGGTGAAAACTGGTCAATAAGCCATATCCTTTATGATTAGATATTCTAGTAATCTGGCATGAAATACACAAATATAATAGCGAGTGATTAGTTTTATATAAGTATTTCTATTAAACCTCTAATAATTACCGCTTTACATATAGATTTTTTTAGTTGGATGCGGGCTTTAGTACCATATAGCTTTACCTGAATCTTATCGTTTGCTTTGGCTGATCCCCCAGGATCTGCGACATAATGCCAAACACAAGCAACTTGTTCGCCTGATCTTCGCAATCCCAGCAGGTTAACTACCCGATTTTATCGAGCAACAGCAAACTCTTTGAACTGATTTGCTATATTTCATTTATGGCCGATTCCAAGTCCATGTCCATTTCCCATTTCCATGTTCGTGTCCAAGTCCGTTGTTCGTTGTCCATTTCGATGTCAGTCATTCATTTTTGTGCTCGACTGCTAATGCCTTAAAGCCAAAATCCATTTCGTCGTTTGGCCATATAATCCAACATTATACATTAAATATGGCACTTGAAAGCACCGCGCGTCTGCATGCCAATTTCAAGGCCCCTTTTCCTCCACTTCACCACCCACAGCATACCATCCCATATCCCACCGGAATTTTCCGATTTTCCCCATTTTCCCCCAACAACACCACCACCTTGCTGCGATCGCACTTGTCGCTCGCGCCGGCGCCTTCAGGGTCGTCAATTTTTTGAGCGACATTTCGAAACGGTGGCTAAAATCACAAAAGCCAACAGAAAAAAATAAAAATCAACTATGGCGAAAAAGTAATACAAATGAAATCCGAAAACAAATAAGCGATTTTGTTTGTGGGGAGAAATGATGATGATCGGCGCCAGACGGACATAAAAACGCCAAAAGAAACGGAAAGGAAGCCTGGGAAAGGCGCATTTCCACTTTGTTTATTCGGAGCTCTGAAGCTCTTTTTAATGGTTTCTTTTTTTTTTTTTTTTTGGCCAAATGGCCAATTAATGCGATTTACTTGATACGCTTTTGCATTTTGTTGTTGCTTTACGACGGCGGATATTAATTAATTGGCCCGTCTCTGGGGAGCAATTCCCATTGTGCCCATCaagagtccacctatcatcccagttcgcagtccaaacctga

## Supporting information

S1 Fig Despite changes in *brk* expression patterns in the early embryo, the *brk* enhancer mutants do not exhibit the cuticle phenotypes associated with *brk*- gene mutants. (A) Dark field images of lateral view of cuticle preps from first instar larvae. (B) Bright field images taken with 40X objective of dentical bands in the A2 abdominal segment. Ventral view with anterior to the left.
(TIF)

S2 Fig NanoString reveals changes in expression of pivotal patterning genes in *brk* enhancer mutant embryos. Results for 70 genes assayed by NanoString in nc14C (late stage 5) embryos (see Materials and methods). The results are given in arbitrary units and the SEM for 6 (WT, *brkΔ5’Δ3’*) or 5 (*brkΔ5’*, *brkΔ3’*) individual embryos are shown by black bars. WT shown in black, *brkΔ5’* in gold, *brkΔ3’* in blue and *brkΔ5’Δ3’* in grey. Genes broken into groups based on expression level for ease of display.
(TIFF)

S3 Fig Brk plays a role in regulating mesodermal gene expression in late stage 5 embryos. (A-B) Screen shots from database of Brk ChIP-seq data [28] showing binding of Brk in early stage 5 (2–2.5hr) and late stage 5 (3–3.5 hr) to the (A) *netA* and (B) *stumps* gene loci. Brk binding is significant (i.e. peak calls shown as black boxes under tracks) only in late stage 5. Flybase defined protein coding regions for each gene shown in blue under Brk ChIP-seq tracks.
(TIF)

S4 Fig Effects on BMP regulators and target genes varies between the *brk* mutant lines. (A-C) Dorsal view of stage 6 embryos hybridized with riboprobes to (A) *tsg* and (B) *vn*, or immunostained with an antibody to (C) dpErk. (D) Cross section of stage 6 embryos showing *zen* expression (green). Magnified image shows only dorsal one-third of embryo. Representative images for each genotype, further quantified in E. (E) Box plot of width, in number of cells, expressing *zen*. P-values determined by Welsh’s t-test comparing *brkΔ5’*, *brkΔ3’*, and *brkΔ5’Δ3’* to WT for *zen* were P = 0.4, P = 5.5x10^-5^, P = 0.06, respectively. Significance indicated in graph by *P<0.05, ***P<0.0001. (F) Percentage of embryos showing normal (blue) vs disrupted (orange) expression of *Race* in early stage 6 embryos. Number of embryos counted for each graph in this figure indicated under genotype.
(TIF)

S5 Fig Brk is involved in canalizing amnioserosa and directly affects the expression of key components of the canalization network. (A) FISH staining of early stage 5 embryos, lateral views, with riboprobes to *brk* and *egr*. *egr* expression is diminished or lost in the *brkΔ5’ a*nd *brkΔ5’Δ3’* embryos. (B) *In situ* hybridization of late stage 5 embryos, dorsal views, with riboprobes to *egr*. *egr* expression remains low in the *brkΔ5’* but is expanded in the *brkΔ3’* embryos. (C) FISH staining of late stage 5 embryos, lateral views, with riboprobes to *Cv-2*. White arrows indicate expanded *Cv-2* expression in the *brkΔ5’ a*nd *brkΔ5’ Δ3’* embryos. (D) Model of canalization loop acting to regulate amnioserosa cell number, reproduced from [45]. (E-G) Screen shots from database of Brk ChIP-seq data [28] showing binding of Brk in early stage 5 (2–2.5hr) and late stage 5 (3–3.5 hr) to the (E) *egr*, (F) *Cv-2*, and (G) *zen* loci.
(TIF)

S6 Fig Changes in dorsal-lateral gene expression and amnioserosa cell number in *brk* CRISPR mutants is specific to changes in brk expression. (A-E) FISH staining of late stage 5 embryos, lateral views, with riboprobes to *tld* (green), *ind* and *sna* (both purple). All embryos are trans-heterozygous females of the genotypes indicated. Consistent with the patterns seen in the homozygous *brk* CRISPR mutants, *tld* is expanded ventrally, beyond the domain of *ind* expression in the trans-heterozygous embryos with *brkΔ5’ a*nd *brkΔ5’ Δ3’* but not significantly in *brkΔ3’*. (F) Comparison of number of amnioserosa cells in homozygous *brk* enhancer mutants to trans-heterozygous combinations with *brk-* gene mutant. Homozygous mutant data is reproduced from Fig 1 and placed next to the trans-heterozygous data for comparison.
(TIF)

S1 Dataset Numerical data associated with each graph. Excel file containing raw counts for all graphically represented data depicted in Figs 1, 2, 4, 5 and 7, S1 Fig, S2 Fig, S4 Fig, and S6 Fig.
(XLSX)
