# Supplementary figures and images for: Coacting enhancers can have complementary functions within gene regulatory networks and promote canalization
Source: PLoS Genet. 2019 Dec 12;15(12):e1008525. doi: 10.1371/journal.pgen.1008525 (PMC6932828; doi:10.1371/journal.pgen.1008525)

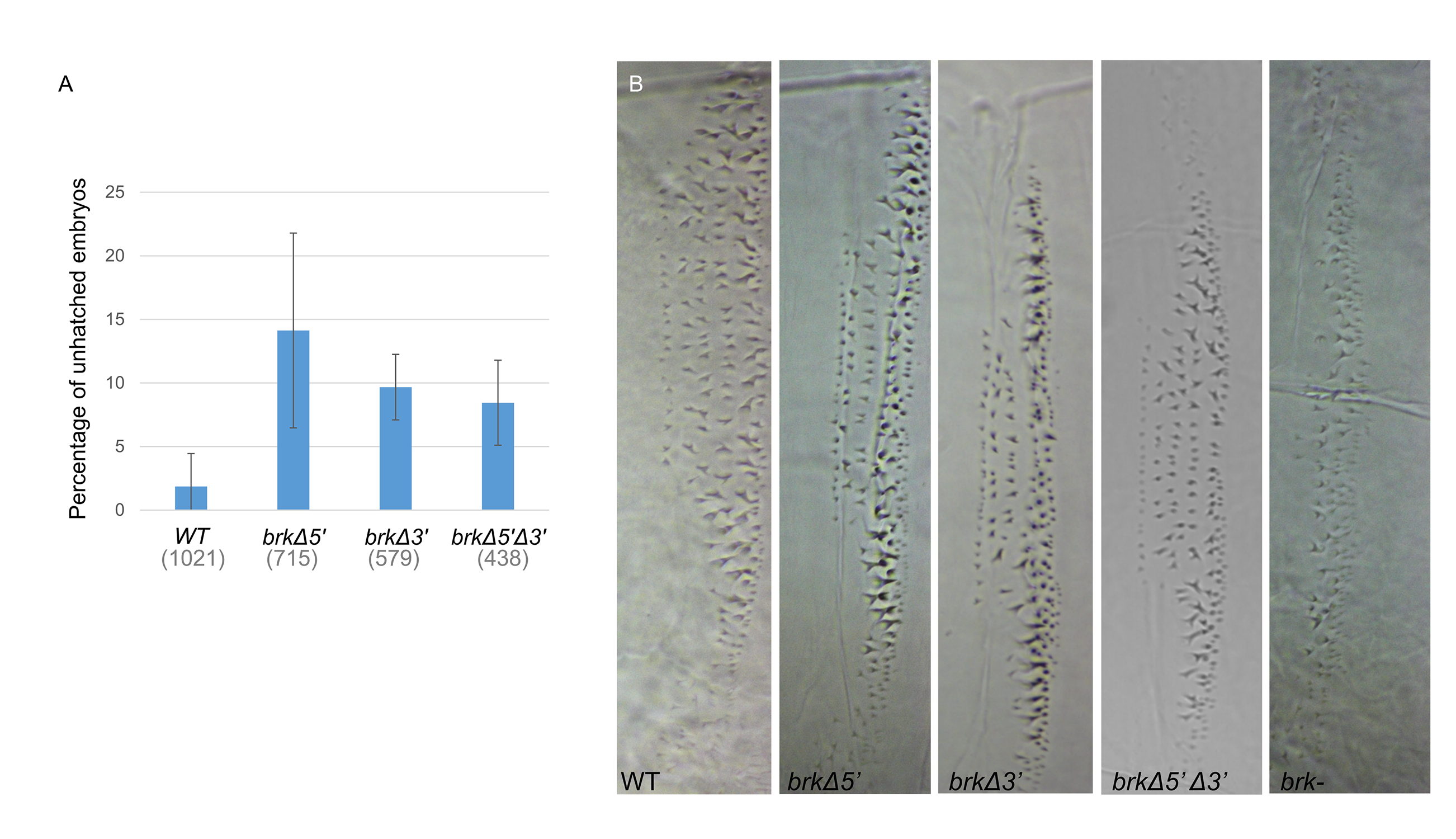

Supplement: S1 Fig — (A) Dark field images of lateral view of cuticle preps from first instar larvae. (B) Bright field images taken with 40X objective of dentical bands in the A2 abdominal segment. Ventral view with anterior to the left. (TIF) [file pgen.1008525.s001.tif]

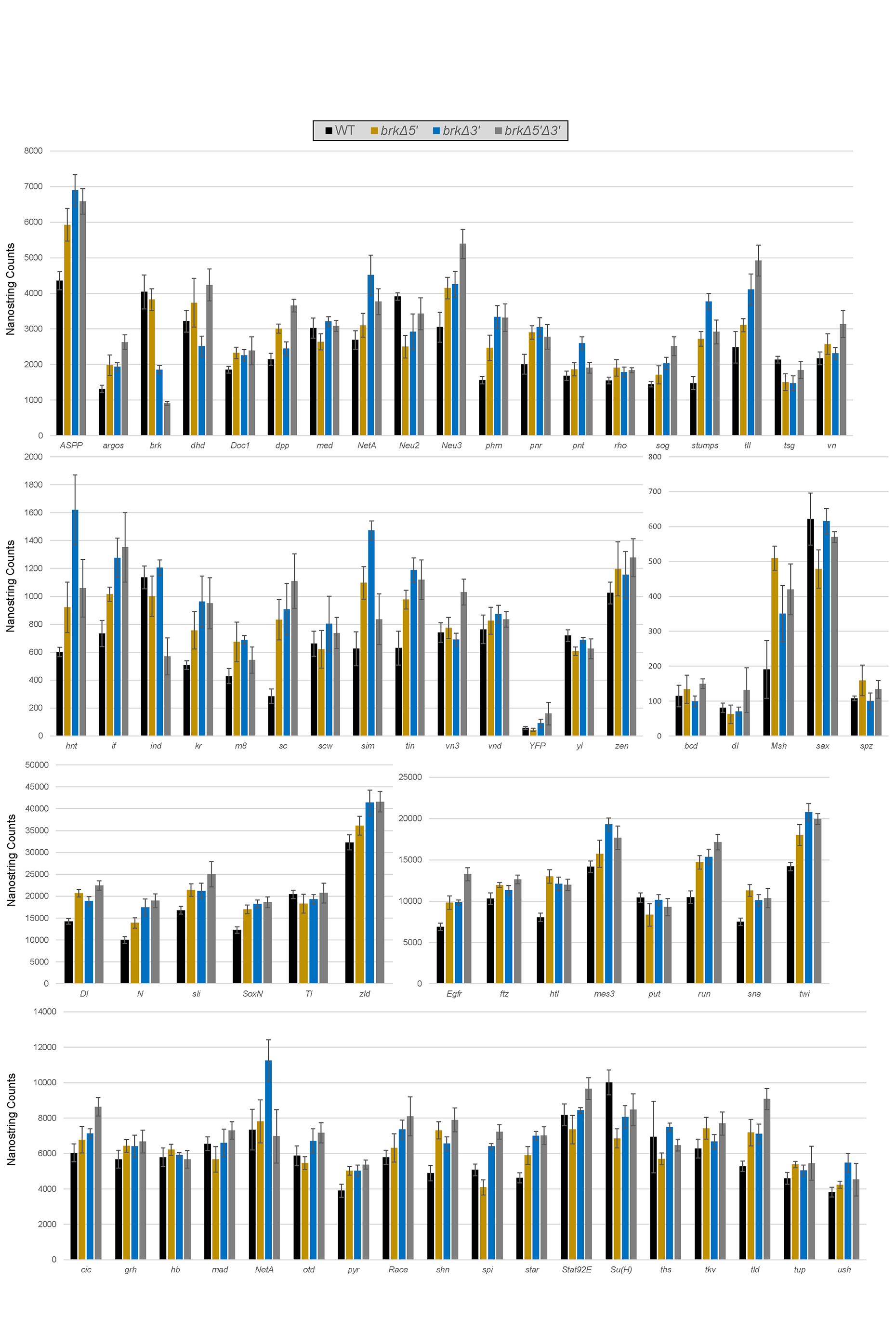

Supplement: S2 Fig — Results for 70 genes assayed by NanoString in nc14C (late stage 5) embryos (see Materials and methods). The results are given in arbitrary units and the SEM for 6 (WT, brkΔ5’Δ3’) or 5 (brkΔ5’, brkΔ3’) individual embryos are shown by black bars. WT shown in black, brkΔ5’ in gold, brkΔ3’ in blue and brkΔ5’Δ3’ in grey. Genes broken into groups based on expression level for ease of display. (TIFF) [file pgen.1008525.s002.tiff]

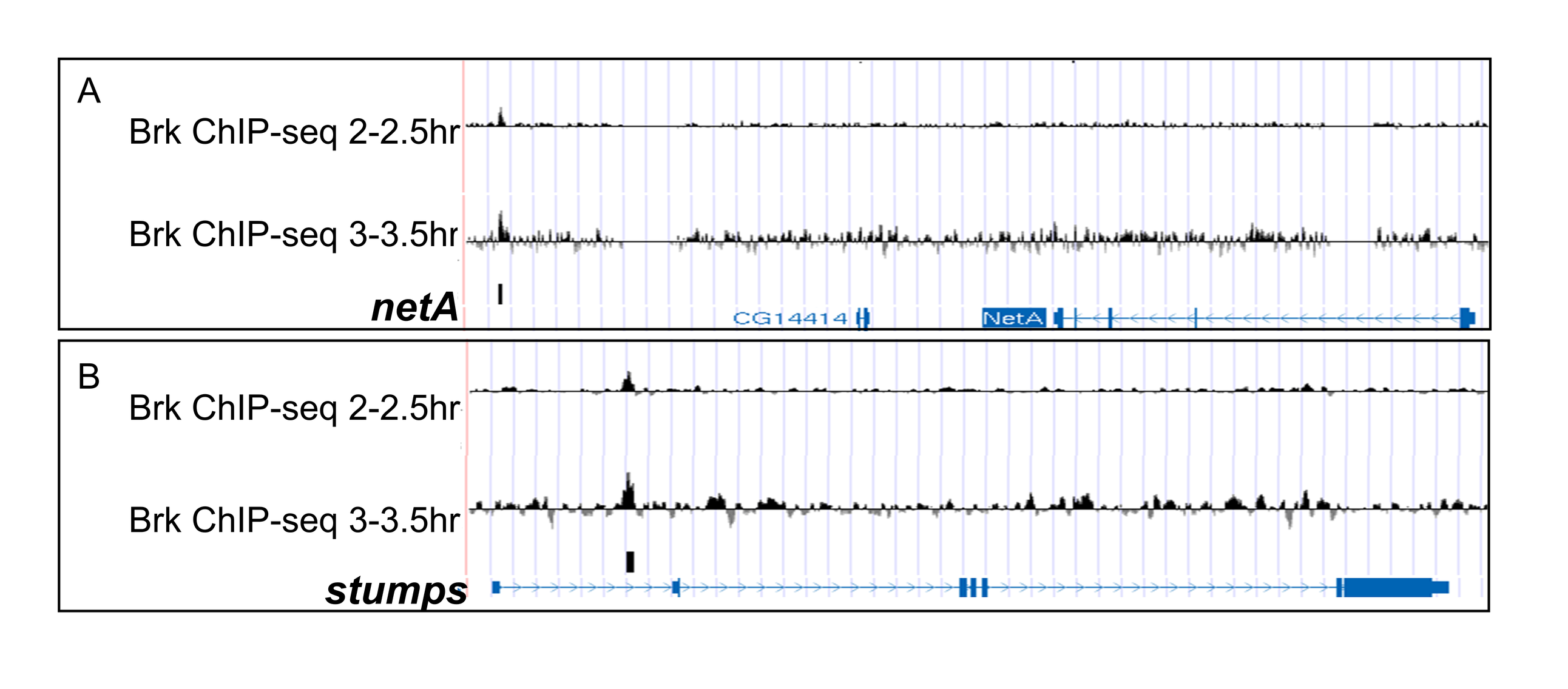

Supplement: S3 Fig — (A-B) Screen shots from database of Brk ChIP-seq data [28] showing binding of Brk in early stage 5 (2–2.5hr) and late stage 5 (3–3.5 hr) to the (A) netA and (B) stumps gene loci. Brk binding is significant (i.e. peak calls shown as black boxes under tracks) only in late stage 5. Flybase defined protein coding regions for each gene shown in blue under Brk ChIP-seq tracks. (TIF) [file pgen.1008525.s003.tif]

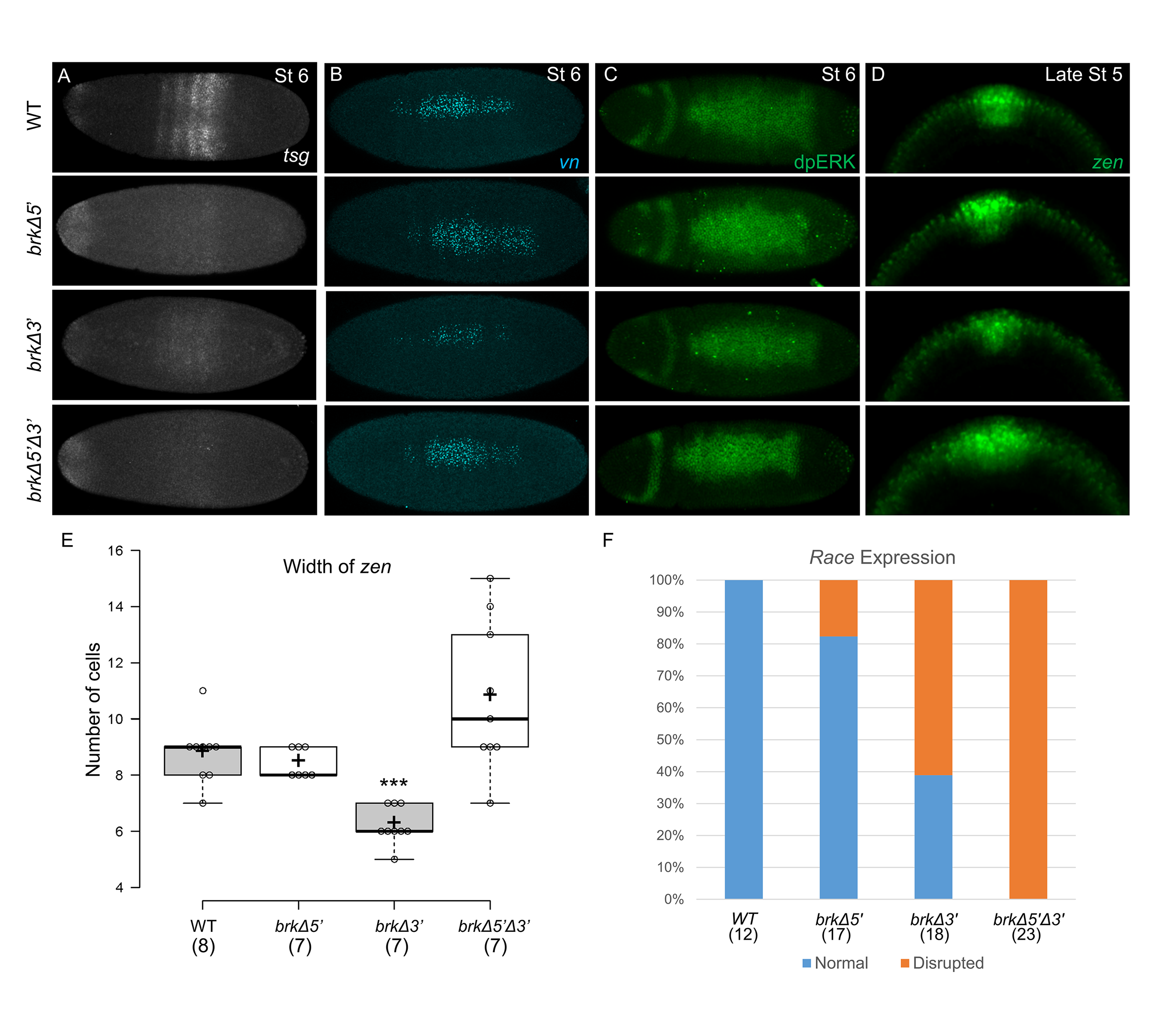

Supplement: S4 Fig — (A-C) Dorsal view of stage 6 embryos hybridized with riboprobes to (A) tsg and (B) vn, or immunostained with an antibody to (C) dpErk. (D) Cross section of stage 6 embryos showing zen expression (green). Magnified image shows only dorsal one-third of embryo. Representative images for each genotype, further quantified in E. (E) Box plot of width, in number of cells, expressing zen. P-values determined by Welsh’s t-test comparing brkΔ5’, brkΔ3’, and brkΔ5’Δ3’ to WT for zen were P = 0.4, P = 5.5x10^-5^, P = 0.06, respectively. Significance indicated in graph by *P<0.05, ***P<0.0001. (F) Percentage of embryos showing normal (blue) vs disrupted (orange) expression of Race in early stage 6 embryos. Number of embryos counted for each graph in this figure indicated under genotype. (TIF) [file pgen.1008525.s004.tif]

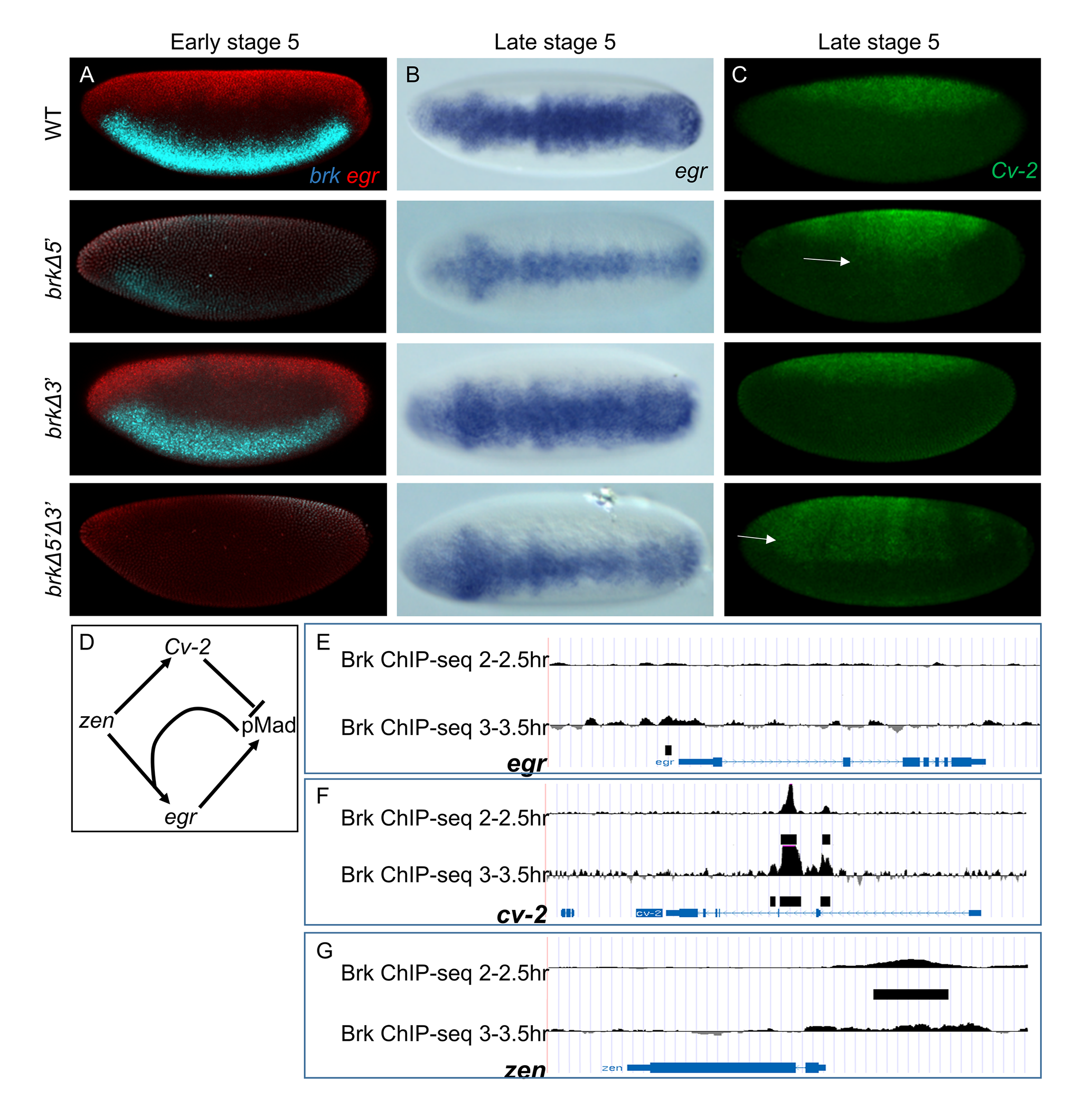

Supplement: S5 Fig — (A) FISH staining of early stage 5 embryos, lateral views, with riboprobes to brk and egr. egr expression is diminished or lost in the brkΔ5’ and brkΔ5’Δ3’ embryos. (B) In situ hybridization of late stage 5 embryos, dorsal views, with riboprobes to egr. egr expression remains low in the brkΔ5’ but is expanded in the brkΔ3’ embryos. (C) FISH staining of late stage 5 embryos, lateral views, with riboprobes to Cv-2. White arrows indicate expanded Cv-2 expression in the brkΔ5’ and brkΔ5’ Δ3’ embryos. (D) Model of canalization loop acting to regulate amnioserosa cell number, reproduced from [45]. (E-G) Screen shots from database of Brk ChIP-seq data [28] showing binding of Brk in early stage 5 (2–2.5hr) and late stage 5 (3–3.5 hr) to the (E) egr, (F) Cv-2, and (G) zen loci. (TIF) [file pgen.1008525.s005.tif]

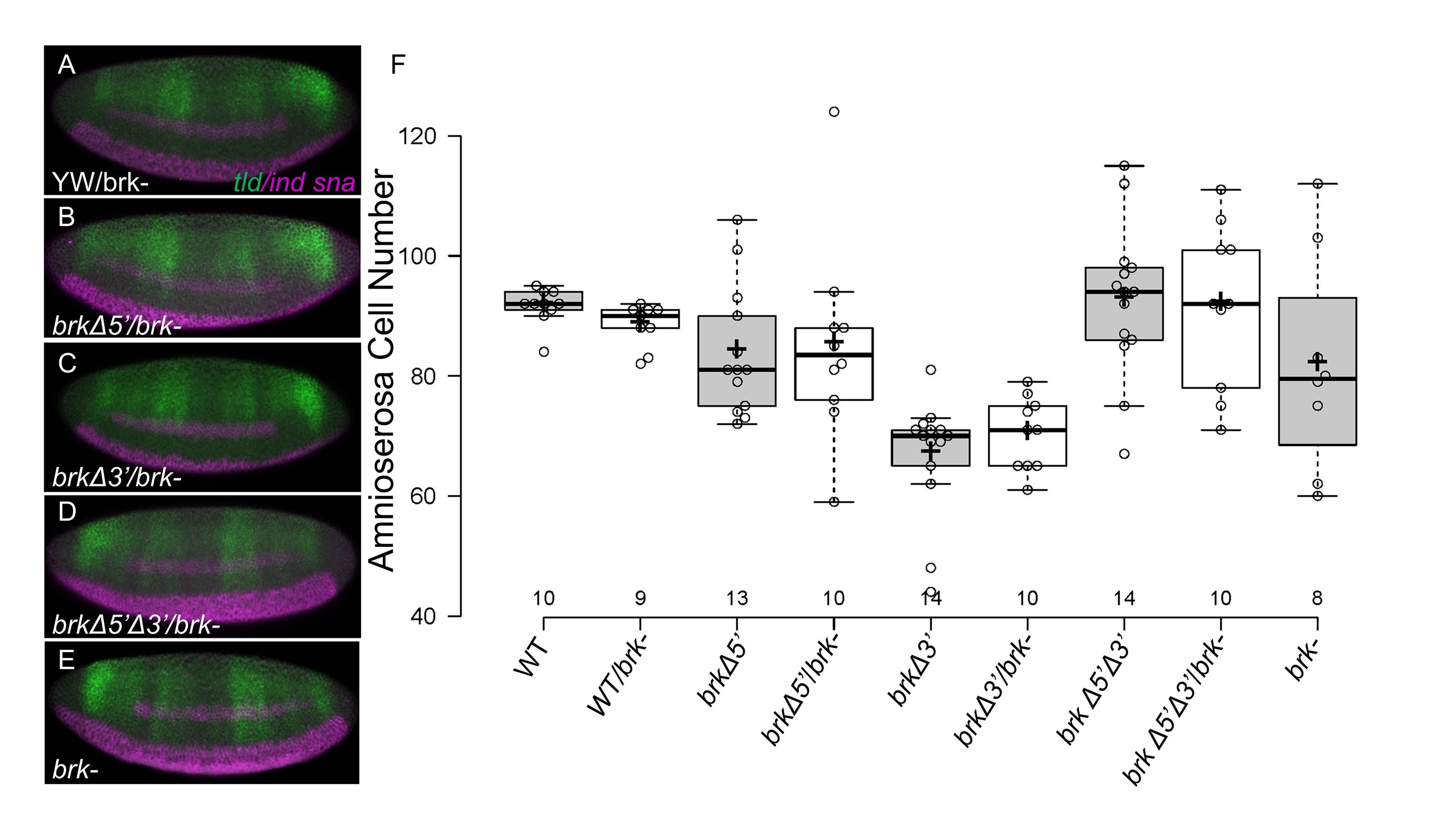

Supplement: S6 Fig — (A-E) FISH staining of late stage 5 embryos, lateral views, with riboprobes to tld (green), ind and sna (both purple). All embryos are trans-heterozygous females of the genotypes indicated. Consistent with the patterns seen in the homozygous brk CRISPR mutants, tld is expanded ventrally, beyond the domain of ind expression in the trans-heterozygous embryos with brkΔ5’ and brkΔ5’ Δ3’ but not significantly in brkΔ3’. (F) Comparison of number of amnioserosa cells in homozygous brk enhancer mutants to trans-heterozygous combinations with brk- gene mutant. Homozygous mutant data is reproduced from Fig 1 and placed next to the trans-heterozygous data for comparison. (TIF) [file pgen.1008525.s006.tif]
